# Impairment of Immunoproteasome Function by β5i/LMP7 Subunit Deficiency Results in Severe Enterovirus Myocarditis

**DOI:** 10.1371/journal.ppat.1002233

**Published:** 2011-09-01

**Authors:** Elisa Opitz, Annett Koch, Karin Klingel, Frank Schmidt, Stefan Prokop, Anna Rahnefeld, Martina Sauter, Frank L. Heppner, Uwe Völker, Reinhard Kandolf, Ulrike Kuckelkorn, Karl Stangl, Elke Krüger, Peter M. Kloetzel, Antje Voigt

**Affiliations:** 1 Medizinische Klinik für Kardiologie, Charité-Universitätsmedizin Berlin, Berlin, Germany; 2 Institut für Biochemie, Charité-Universitätsmedizin Berlin, Berlin, Germany; 3 Abteilung Molekulare Pathologie, Institut für Pathologie und Neuropathologie, Eberhard-Karls-Universität, Tuebingen, Germany; 4 Interfakultäres Institut für Genetik und Funktionelle Genomforschung, Ernst-Moritz-Arndt-Universität, Greifswald, Germany; 5 Institut für Neuropathologie, Charité-Universitätsmedizin Berlin, Berlin, Germany; Columbia University, United States of America

## Abstract

Proteasomes recognize and degrade poly-ubiquitinylated proteins. In infectious disease, cells activated by interferons (IFNs) express three unique catalytic subunits β1i/LMP2, β2i/MECL-1 and β5i/LMP7 forming an alternative proteasome isoform, the immunoproteasome (IP). The *in vivo* function of IPs in pathogen-induced inflammation is still a matter of controversy. IPs were mainly associated with MHC class I antigen processing. However, recent findings pointed to a more general function of IPs in response to cytokine stress. Here, we report on the role of IPs in acute coxsackievirus B3 (CVB3) myocarditis reflecting one of the most common viral disease entities among young people. Despite identical viral load in both control and IP-deficient mice, IP-deficiency was associated with severe acute heart muscle injury reflected by large foci of inflammatory lesions and severe myocardial tissue damage. Exacerbation of acute heart muscle injury in this host was ascribed to disequilibrium in protein homeostasis in viral heart disease as indicated by the detection of increased proteotoxic stress in cytokine-challenged cardiomyocytes and inflammatory cells from IP-deficient mice. In fact, due to IP-dependent removal of poly-ubiquitinylated protein aggregates in the injured myocardium IPs protected CVB3-challenged mice from oxidant-protein damage. Impaired NFκB activation in IP-deficient cardiomyocytes and inflammatory cells and proteotoxic stress in combination with severe inflammation in CVB3-challenged hearts from IP-deficient mice potentiated apoptotic cell death in this host, thus exacerbating acute tissue damage. Adoptive T cell transfer studies in IP-deficient mice are in agreement with data pointing towards an effective CD8 T cell immune. This study therefore demonstrates that IP formation primarily protects the target organ of CVB3 infection from excessive inflammatory tissue damage in a virus-induced proinflammatory cytokine milieu.

## Author Summary

The proteasome recognizes and degrades protein substrates tagged with poly-ubiquitin chains. Immune cells and cells activated by inflammatory cytokines/interferons express immunoproteasomes (IPs) that are characterized by unique catalytic subunits with increased substrate turnover. In infectious disease, the function of IPs is still a matter of controversial debate. Here, we report on a novel innate function of IPs in viral infection. We studied the murine model of acute enterovirus myocarditis, which represents one of the most common viral disease entities among young people. We found that IPs protect the pathogen-challenged tissue from severe injury, which was reflected in severe myocardial destruction and large inflammatory foci in mice lacking IPs. We show data that this prevention of excessive inflammatory tissue damage in viral heart disease is primarily attributed to preservation of protein homeostasis due to accelerated substrate turnover by IPs. Thus, a major innate function of IPs in viral infection is to stabilize cell viability in inflammatory tissue injury.

## Introduction

Unfolded or misfolded proteins are potentially harmful to cells and have to be efficiently eliminated before they intoxicate the intracellular environment. This is of particular importance during proteotoxic stress as a consequence of intrinsic or extrinsic factors when the levels of misfolded proteins are transiently or persistently elevated (Dantuma, 2010 #1). In viral infection cytokine exposure and inflammation induce the generation of reactive oxygen species in both immune and non-immune cells [1], [2] with concomitant oxidant-protein damage and proteotoxic stress. An important defence mechanism is the specific destruction of these proteins by the ubiquitin-proteasome system (UPS) [3]. The UPS is among others involved in the regulation of protein quality control in cardiovascular pathologies [4]–[6], in neurodegenerative disorders and other human pathologies [7], [8]. The UPS with the 26S proteasome as central proteolytic unit represents the major ATP-dependent degradation system in eukaryotes responsible for the maintenance of protein homeostasis and the generation of the vast majority of antigenic peptides that are presented by MHC class I molecules to CD8^+^ T cells in infectious disease [9]. Short-lived regulatory proteins involved in cell differentiation, cell-cycle regulation, transcriptional regulation, or apoptosis, but also aberrant proteins are directed to proteasomal degradation through conjugation with the small protein modifier ubiquitin via a cascade of E1, E2, and E3 enzymes, thus forming poly-ubiquitinylated (poly-ub) proteins [10]. Poly-ub proteins are substrates for 26S proteasomes which are formed through the association of two 19S regulator complexes with the catalytic core complex, the 20S proteasome, that hydrolyzes proteins into shorter peptide fragments [11], [12]. Peptide hydrolyzing activity of the 20S core is restricted to three β-subunits, i.e. β1, β2, and β5, located in the two inner heptameric β-rings of the 20S proteasome [13].

Upon interferon (IFN)-exposure of cells or tissues, alternative catalytically active β subunits, i.e. β1i/LMP2, β2i/MECL-1, and β5i/LMP7, are induced. These so called immunosubunits are incorporated into newly formed 20S immunoproteasomes (IP) in a process that is driven by β5i/LMP7 [14]. β1i/LMP2 and β5i/LMP7 are encoded within the major histocompatibility II region and their incorporation into IPs induces altered proteolytic characteristics that result in many cases in more efficient liberation of MHC class I epitopes [15]–[17] particularly within the early phase of antiviral immunity [18], [19]. This increase in MHC class I peptide supply by IPs appears to be important for triggering an effective early CD8 T cell response [20]–[23]. However, controversial findings about the association between IP function and CD8 T cell priming raised some doubts with regard to the *in vivo* impact of these data. In fact, an alternative physiological function of IPs has been demonstrated recently by our group in that IPs protect cells against cytokine induced oxidative damage, thus preserving protein homeostasis. Substrate modification of oxidant-damaged proteins with poly-ubiquitin results in protein degradation particularly by IPs [24]. Nevertheless, conclusive studies investigating the role of IP in response to viral infection beyond the analysis of specific T cell immunity have not been performed. Also, the importance of this regulated protease in cardiac disease remains to be elucidated. Within the context of the murine model of ongoing coxsackievirus B3 (CVB3)-myocarditis, we recently reported on cardiac IP formation early upon infection in mice being resistant to chronic disease. The remarkably delayed induction of cardiac IPs in susceptible mice pointed towards a potential disease-modifying effect of this finding [19].

Here, we show that cardiac IP prevent exacerbation of acute CVB3-induced myocardial destruction and possess a protective function in viral heart disease expanding their role to the protection of cells against inflammation induced toxic effects thereby stabilizing cell viability in viral infection.

## Results

### Characterization of cardiac 20S proteasomes in CVB3-myocarditis

**Figure 1:**
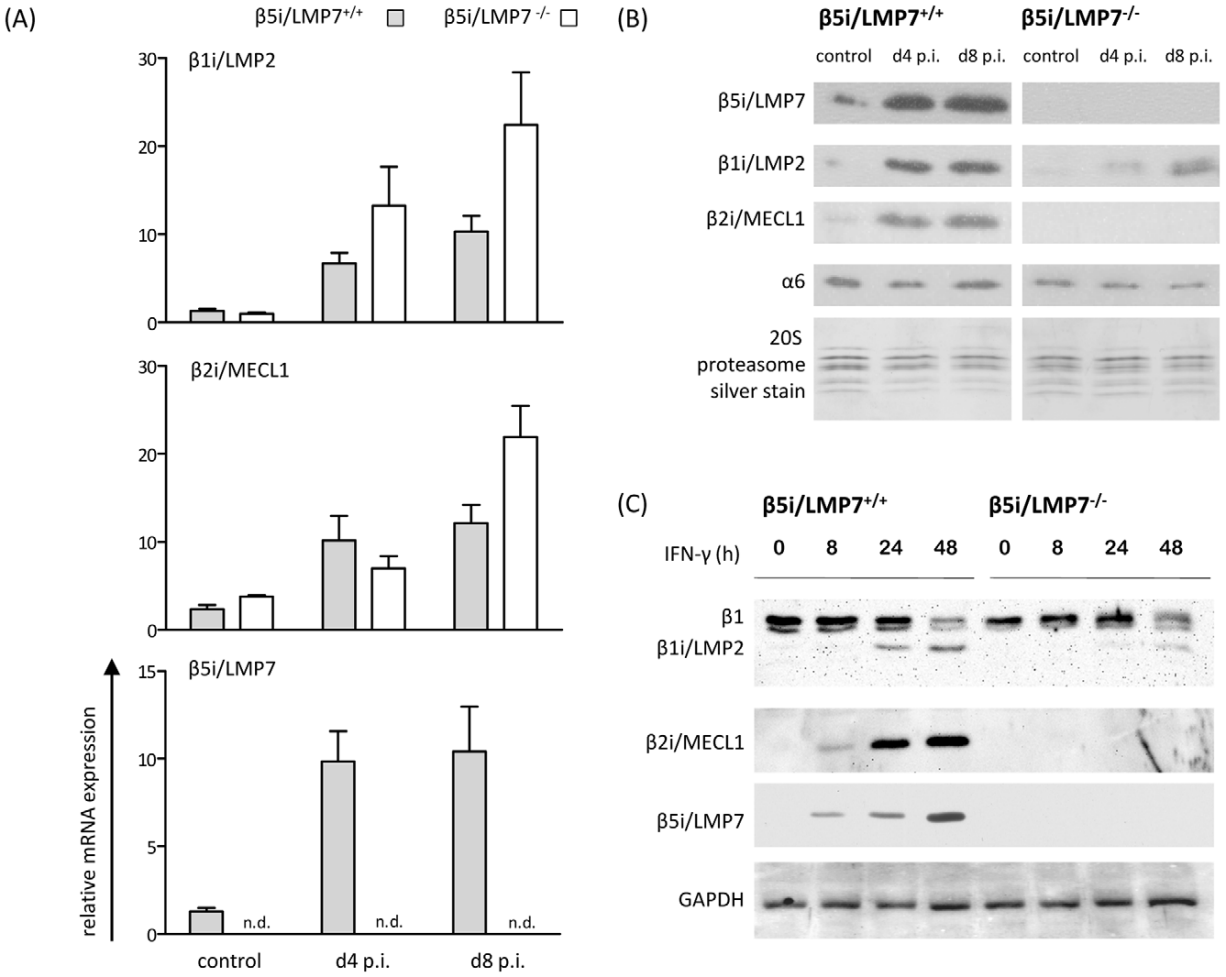
Characterization of 20S proteasomes in IP-deficient mice in CVB3-myocarditis. (A) Quantitative real-time PCR: β1i/LMP2, β2i/MECL-1, and β5i/LMP7 mRNA expression in the hearts of β5i/LMP7^+/+^ and β5i/LMP7^-/-^ mice at d4 and d8 p.i. Data shown are representative means of n = 5 mice±SEM. (B) 20S proteasomes were isolated from cardiac tissue and subjected to SDS-PAGE followed by immunoblotting (proteasome subunit α6 is a loading control). Silver staining of isolated 20S proteasomes illustrates equal loading. Data shown are representative for two different 20S proteasome purifications (n = 10 mice per experiment). (C) Primary embryonic cardiomyocytes were stimulated with 100 U/ml IFN-γ for indicated time points and the expression of 20S proteasome immunosubunits was determined in whole tissue homogenates. β1i/LMP2 antibody shows a cross-reaction to constitutive β1 subunit. GAPDH illustrates equal protein loading.

One well-established model to study myocardial inflammation is the induction of murine myocarditis with coxsackievirus B3 (CVB3) in C57BL/6 mice leading to acute heart muscle injury at d8 p.i. [19]. Here, we have challenged both β5i/LMP7^+/+^ and β5i/LMP7^-/-^ mice on a C57BL/6 background with CVB3. Cardiac 20S proteasomes isolated from naive mice contain only very small amounts of β1i/LMP2, β5i/LMP7 and β2i/MECL-1 [19], [25]. To test whether β5i/LMP7 deficiency was indeed sufficient to negatively affect the incorporation of all three inducible catalytic subunits into cardiac IP *in vivo*, heart 20S proteasomes were isolated from naive mice and from CVB3-infected mice at the early stage of disease (d4 p.i.) and at the acute stage of myocarditis (d8 p.i.) from both β5i/LMP7^+/+^ and β5i/LMP7^-/-^ mice. Whereas mRNA expression of β1i/LMP2 and β2i/MECL-1 was induced in both hosts (Fig. 1A), immunoblot analysis revealed strongly impaired incorporation of both β1i/LMP2 and β2i/MECL-1 into cardiac 20S proteasomes in acute myocarditis in β5i/LMP7^-/-^ mice (Fig. 1B). Likewise, cytokine stimulation of primary cardiomyocytes from IP-competent mice with IFN-γ resulted in the efficient induction of all three IP subunits, whereas as expected incorporation of β1i/LMP2, β2i/MECL-1 and β5i/LMP7 was impaired in cardiomyocytes isolated from IP-deficient mice (Fig. 1C).

To obtain quantitative information on the content of each proteasome subunit within the 20S core complex *in vivo*, 20S proteasomes were subjected to reverse phase nano HPLC and LTQ-Orbitrap mass spectrometry (MS) analysis. All three inducible subunits β1i/LMP2 (3.5-fold induction; p<0.05), β2i/MECL-1 (2.6-fold induction; p<0.05) and β5i/LMP7 (2.0-fold induction; p = 0.10) were enhanced in CVB3-challenged hearts as early as at d4 p.i. in β5i/LMP7^+/+^ mice. In contrast, no improved incorporation of either β1i/LMP2 (1.3-fold; p = 0.67) or β2i/MECL-1 (1.5-fold; p = 0.38) was observed in cardiac 20S proteasomes from β5i/LMP7^-/-^ mice at this stage of disease. Table 1 indicates relative quantitative expression levels of all three inducible subunits in cardiac proteasomes in wildtype mice with respect to IP-deficient mice. No significant differences were detected in the content of constitutive proteasome subunits as exemplarily shown in Table 1 for catalytic subunits β1, β2, and β5, and for constitutive non-catalytic subunits α2 and β4, respectively. Notably, in agreement with previous studies [26], β5i/LMP7^-/-^ mice show baseline deficits in cardiac β2i/MECL-1 incorporation being aggravated during inflammation (Table 1). Therefore, β5i/LMP7^-/-^ mice encounter a substantial impairment of IP assembly in inflammation-challenged hearts. This appears to be crucial for the interpretation of our data in terms of IP deficiency showing a severe impairment in the incorporation of all three inducible subunits in acute disease *in vivo*.

**Table 1:** Quantification of proteasome subunits by mass spectroscopy in IP-deficient mice.

β5i/LMP7^+/+^/ β5i/LMP7^-/-^	Control		d4 p.i.		d8 p.i.	
	Ratio	p-value	Ratio	p-value	Ratio	p-value
α2	1.18	0.51	1.01	0.99	1.03	0.93
β4	1.17	0.51	1.06	0.84	1.12	0.68
β1	1.19	0.48	1.18	0.63	1.10	0.72
β2	1.37	0.29	1.27	0.50	1.08	0.77
β5	1.12	0.71	0.96	0.91	0.76	0.41
**β5i/LMP7**	**n.d.**		**n.d.**		**n.d.**	
**β1i/LMP2**	**1.32**	**0.48**	**3.70**	**0.04**	**1.82**	**0.18**
**β2i/MECL1**	**5.25**	**0.01**	**9.18**	**<0.001**	**2.64**	**<0.001**

20S proteasomes were isolated from murine hearts from control mice and at day 4 and 8 p.i. from β5i/LMP7^+/+^ and β5i/LMP7^-/-^ mice. The mixture of tryptic 20S proteasome peptides was separated prior to mass spectrometric analysis by reverse phase nano HPLC using a Proxeon System; MS-data were generated on an Orbitrap-MS equipped with a nanoelectrospray ion source as described in material and methods. MS ion intensities are shown for individual proteasome subunits as ratios between β5i/LMP7^+/+^ and β5i/LMP7^-/-^ mice. Data are mean±SD from two technical replicates that have been performed each with two independent biological replicates. p<0.05 indicates statistical significance. n.d.: ratio was not determined for β5i/LMP7.

### Exacerbation of CVB3-myocarditis in IP-deficient mice

**Figure 2:**
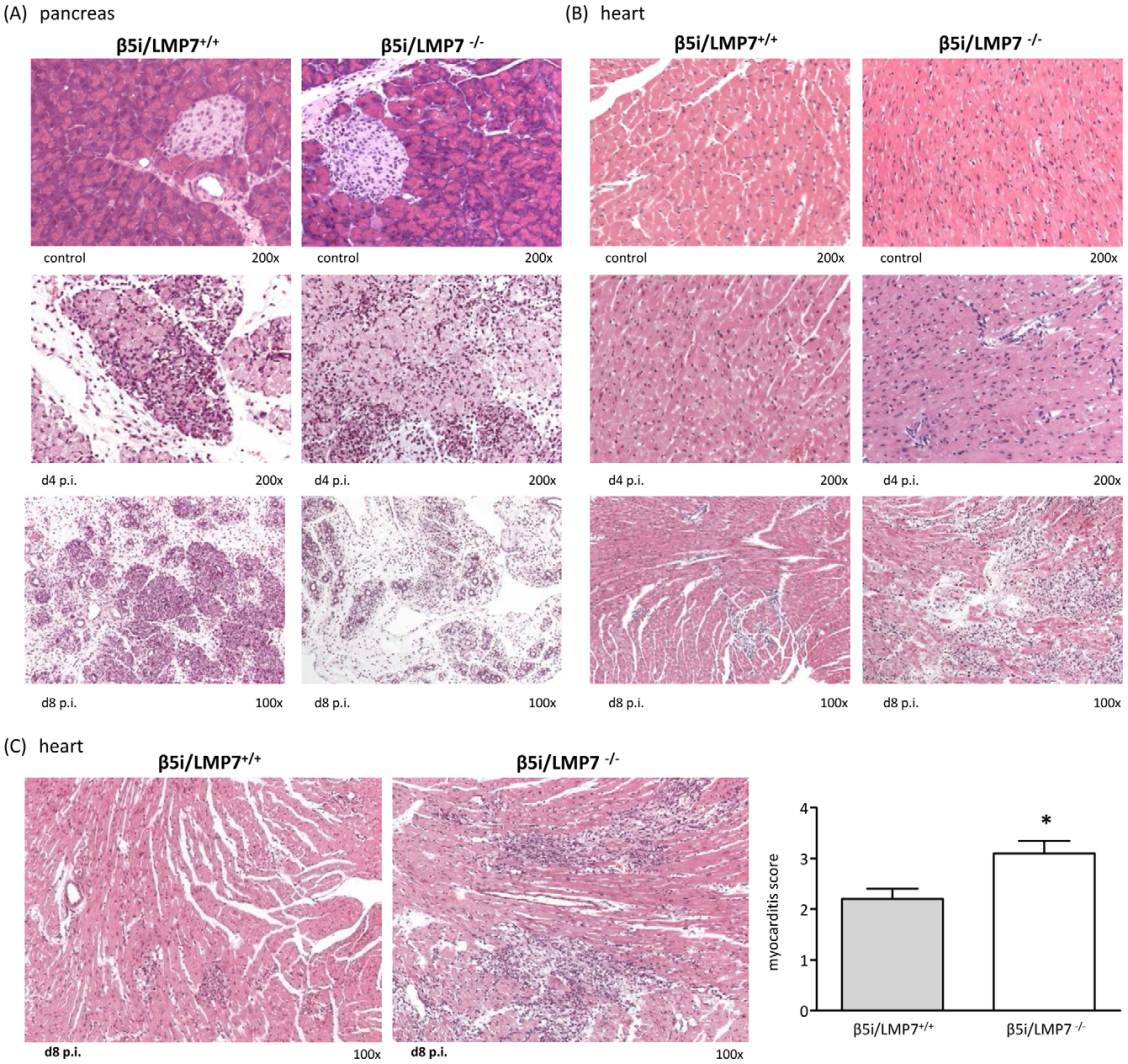
Severe acute CVB3-myocarditis in IP-deficient mice. Representative hematoxylin-eosin (HE)-staining are illustrated from (A) the pancreas and (B) the heart of β5i/LMP7^+/+^ and β5i/LMP7^-/-^ mice at different time points p.i. revealing severe acute myocarditis in β5i/LMP7^-/-^ mice at d8 p.i. All cardiac and pancreatic tissue sections shown are representative for at least n = 10 mice/group. (C) Tissue sections from a different experiment confirmed severe heart muscle injury in β5i/LMP7^-/-^ mice at d8 p.i. (enlarged image). To quantify myocardial damage comprising cardiac cell necrosis, inflammation, and scarring, a myocarditis score from 0 to 4 was applied (0: no inflammatory infiltrates, 1: small foci of inflammatory cells between myocytes, 2: larger foci of >100 inflammatory cells, 3: ≤10% of cross-section involved, 4: 10 to 30% of a cross-section involved) [50] (* p<0.05; mean from n = 5 mice).

Histological analysis of acute myocarditis was done as previously published [27] defining acute myocarditis by lymphocytic infiltrates in association with myocyte necrosis, which we also see in patients with acute myocarditis [28]. CVB3-myocarditis was evaluated in IP-deficient mice at early stages of heart muscle infection (d4 p.i.) and at acute stages of myocarditis (d8 p.i.). Except for some scattered macrophages, no foci of inflammatory infiltrates were observed in the myocardium of both mouse strains at d4 p.i. (Fig. 2B). At this time point severe inflammation of the pancreas, the primary organ of viral replication, was observed in both β5i/LMP7^+/+^ and β5i/LMP7^-/-^ mice (Fig. 2A). Pointing towards accelerated organ destruction in β5i/LMP7^-/-^ mice, representative images of the pancreas at d8 p.i. illustrate final pancreatic islet destruction with fibrous and fatty tissue organ replacement in β5i/LMP7^-/-^ mice, with necrotic cells and massive inflammation still being present in β5i/LMP7^+/+^ mice (Fig. 2A).

Clinically, myocardial tissue damage in CVB3-infection is utmost important since acute myocardial injury may result in severe acute arrhythmia and heart failure. Focussing on myocardial damage comprising cardiac cell necrosis and inflammation in acute myocarditis, IP-deficiency was found to be associated with severe acute myocarditis. As representatively depicted in Fig. 2B and 2C for independent experiments by HE staining, at the acute stage of disease large foci encompassing inflammatory cells and cardiomyocyte necrosis were detected in β5i/LMP7^-/-^ mice, which is in striking contrast to small areas of myocardial inflammation and tissues damage in β5i/LMP7^+/+^ mice. To obtain quantitative information on heart muscle injury, myocarditis scores were determined yielding a score of 3.1±0.3 in β5i/LMP7^-/-^ mice vs. 2.2±0.2 in β5i/LMP7^+/+^ mice (p<0.05, Fig. 2C).

**Figure 3:**
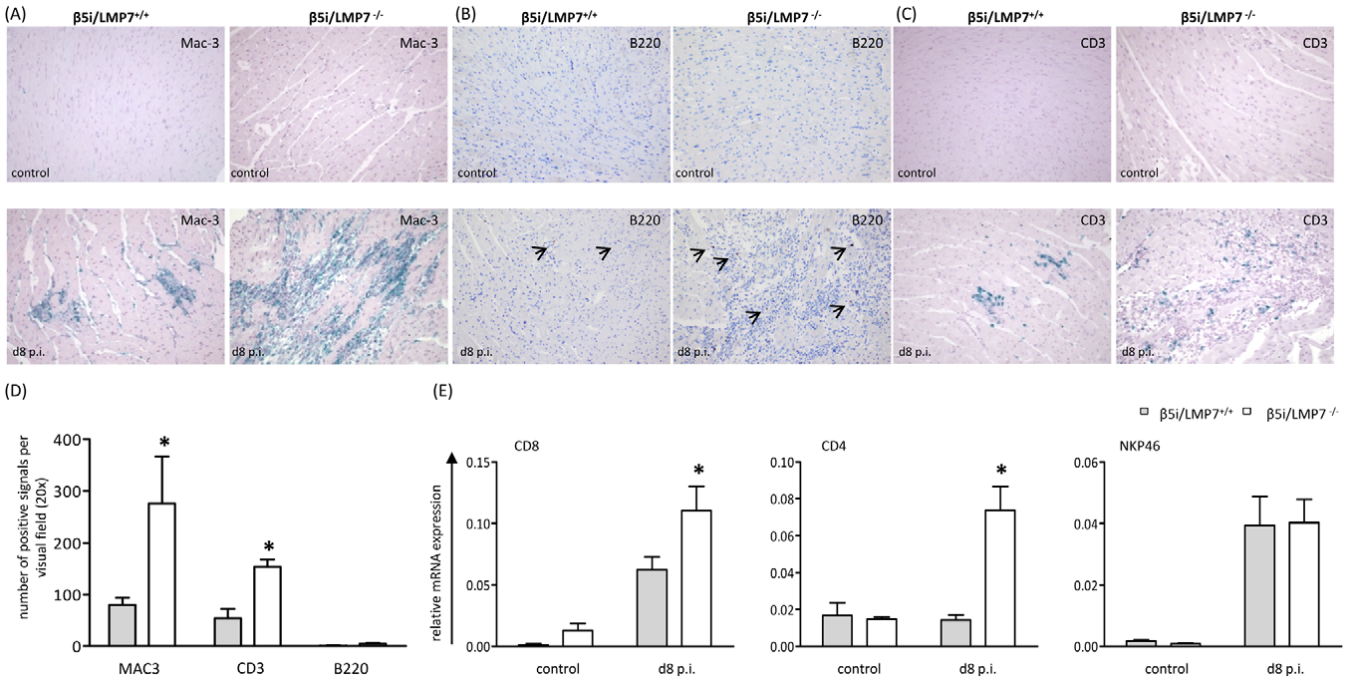
Characterization of myocardial lesions in acute CVB3-myocarditis. To characterize inflammatory lesions in CVB3-infected mice in more detail, immunohistology for (A) Mac-3^+^ macrophages, (B) B220^+^ B lymphocytes and (C) CD3^+^ T lymphocytes were performed. Representative cardiac tissue sections are shown, immunohistology was performed for at least n = 6 mice/group. No specific signals were observed in naive control mice. (D) Quantification of immunohistochemically positive cells was achieved by counting all positive cells per visual field at a magnification of x200 in transverse heart tissue sections. Arithmetic means from n ≥5 mice/group±SEM are shown. Differentiation of individual signals for Mac-3 was limited due to massive macrophage infiltration/staining; thus quantitative data are not reflecting absolute macrophage numbers. (E) Quantitative real-time PCR: mRNA expression of CD8 for CD8 T cells, CD4 for CD4 T cells and NKP46 for natural killer cells was determined in the hearts of β5i/LMP7^+/+^ and β5i/LMP7^-/-^ mice at d8 p.i. Data shown are representative means of n = 5 mice±SEM, * p<0.05.

Macrophages and CD3^+^ T lymphocytes represent the major fraction of invading inflammatory cells in acute CVB3-myocarditis in mice [27], [29]. To address the inflammatory infiltrate in our model in detail, CD3^+^ T lymphocytes, B220^+^ B lymphocytes and Mac-3^+^ macrophages were studied by immunohistology. As demonstrated in Fig. 3, the inflammatory infiltrate was primarily comprised of Mac-3^+^ macrophages and to a lesser extent of CD3^+^ T lymphocytes. B cells were scattered throughout the inflammatory lesions without significantly contributing to the invading cellular infiltrate. In agreement with myocarditis scores (Fig. 2C), quantification of invading cells revealed significantly increased macrophages and T lymphocytes in CVB3-infected β5i/LMP7^-/-^ mice (Fig. 3D). As suggested by quantitative mRNA expression of CD8 and CD4 molecules in the infected myocardium, both CD4^+^ and CD8^+^ T lymphocytes were increased in CVB3-infected β5i/LMP7^-/-^ mice (mRNA expression of CD3 revealed the same result, data not shown). In contrast, expression levels of NKP46, a marker for natural killer (NK) cells, did not differ in both hosts suggesting invasion of NK cells in both hosts to the same extent (Fig. 3E). In summary, immunohistological characterization of myocardial inflammation revealed that cardiac IP formation protected CVB3-challenged hearts from exacerbation of acute heart muscle injury.

**Figure 4:**
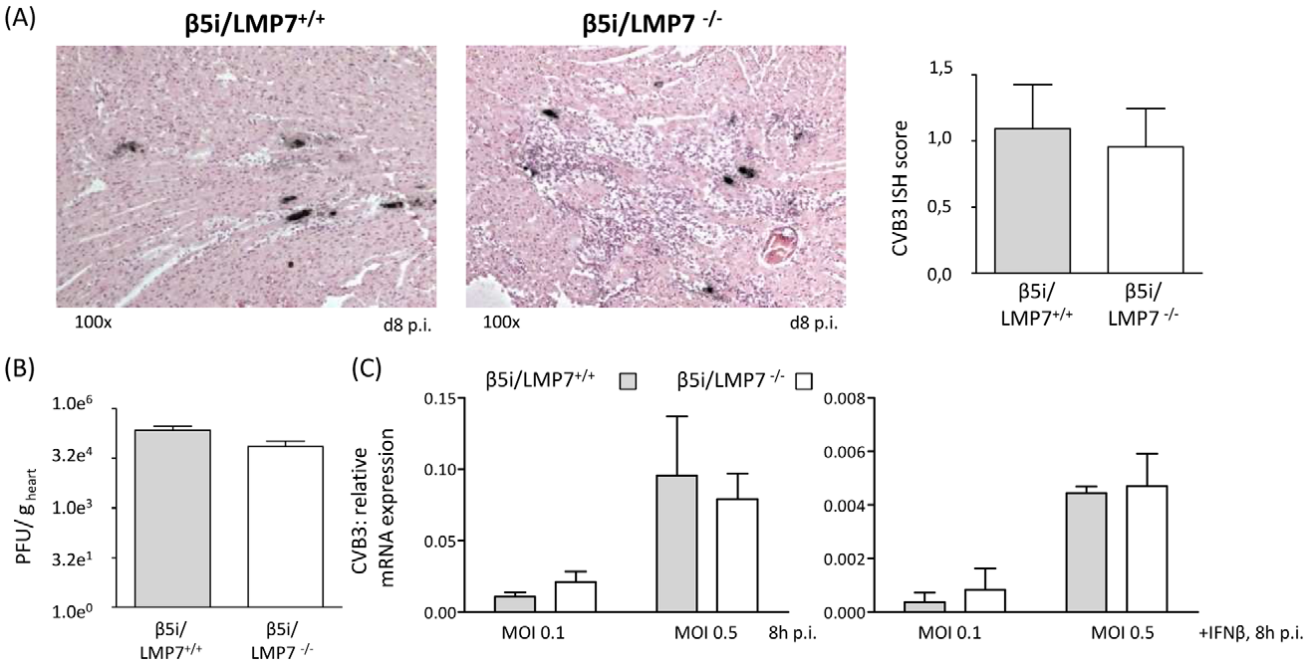
Viral load is not affected in IP-deficient mice. (A) The amount of genomic CVB3 RNA as detected by *in situ* hybridization was scored within a range from 0 to 4 for cardiac tissue sections (n = 10 mice/group). Representative strand-specific *in situ* hybridizations for CVB3 RNA (black dots) in hearts at the acute stage of myocarditis (d8 p.i.) illustrate equal viral load in both hosts. (B) Plaque assays were performed revealing equal amounts of infectious virus particles in CVB3-infected hearts from β5i/LMP7^+/+^ and β5i/LMP7^-/-^ mice (n.s.). (C) Primary cardiomyocytes from β5i/LMP7^+/+^ and β5i/LMP7^-/-^ mice were infected with CVB3 MOI 0.1 and 0.5 for 8 h. To mimic *in vivo* infection, cardiomyocytes were partially cultured with IFN-β for 24 h prior to CVB3 infection. Viral replication was assessed by quantitative real-time PCR. One representative experiment of three independent experiments is shown.

The presence of infected cardiomyocytes adjacent to foci of mononuclear cell infiltrates is pathognomonic in viral myocarditis. Indeed, CVB3 *in situ* hybridization-positive cardiomyocytes were found within inflammatory lesions in acute heart muscle injury in both β5i/LMP7^+/+^ and β5i/LMP7^-/-^ mice (Fig. 4A). However, despite the severity of myocardial tissue damage in CVB3-challenged β5i/LMP7^-/-^ mice, scoring of CVB3 *in situ* hybridization-positive cardiomyocytes pointed towards equal viral replication in both hosts. Also, the titers of cardiac infectious viral particles were found to be within the same range in both β5i/LMP7^+/+^ and β5i/LMP7^-/-^ mice in acute disease (Fig. 4B). To further investigate viral replication within the context of IP-deficiency, primary cardiomyocytes from β5i/LMP7^+/+^ and β5i/LMP7^-/-^ mice were infected with CVB3 *in vitro* and CVB3 replication was determined by quantitative real-time PCR. These experiments were also carried out in the presence of type I IFN-stimulation to mimic the *in vivo* cytokine milieu in acute heart muscle injury. As shown in Fig. 4C, IP-deficiency revealed no influence on CVB3 replication *in vitro*.

**Figure 5:**
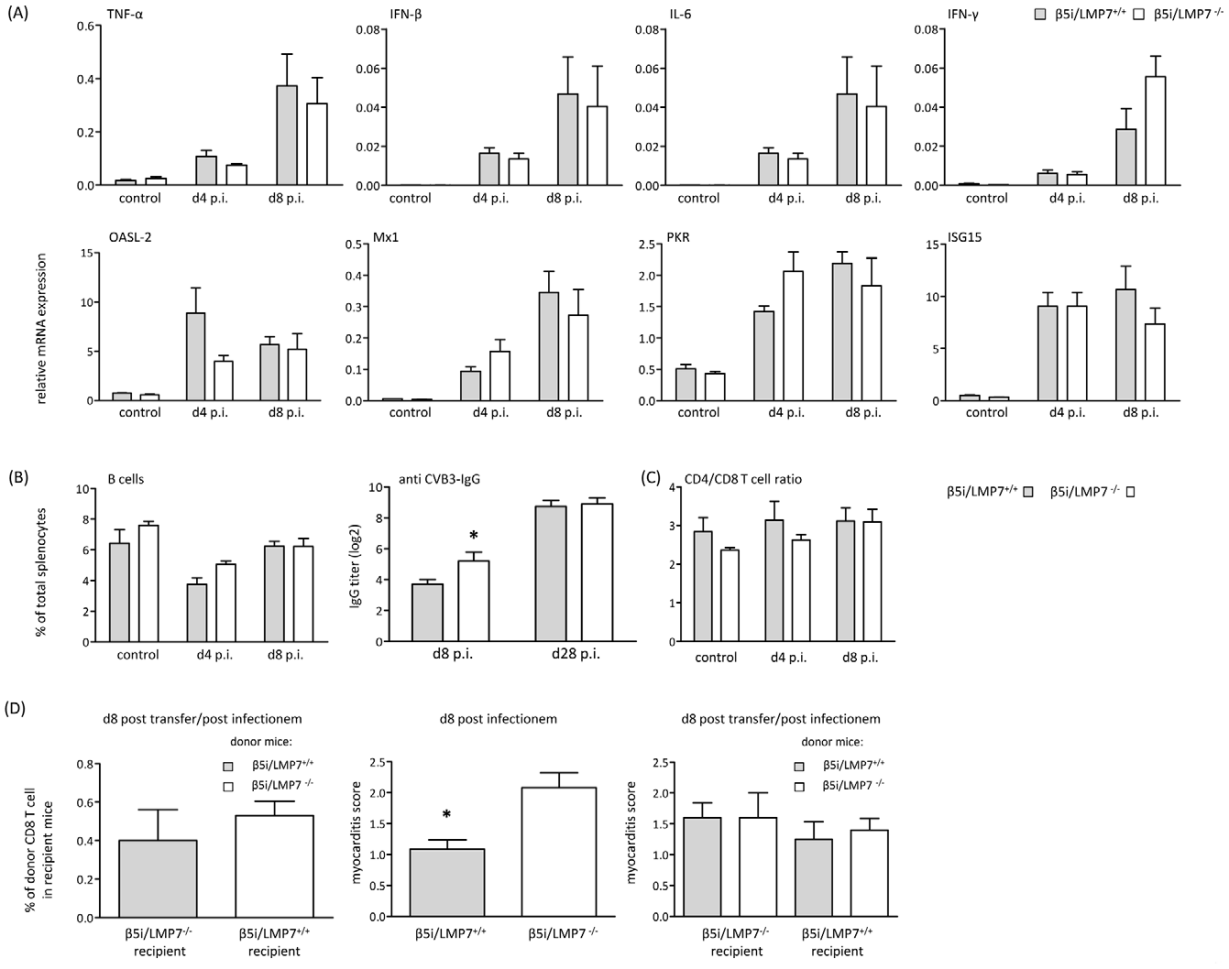
Adaptive immune responses in CVB3-infected IP-deficient mice. (A) Quantitative real-time PCR: mRNA expression of indicated cytokines (first row) and antiviral molecules of the innate immune response (second row) was determined in the hearts of β5i/LMP7^+/+^ and β5i/LMP7^-/-^ mice at d4 and d8 p.i. Data shown are mean of n≥5 mice±SEM. (B)+(C) Flow cytometric analysis was completed on splenocytes isolated from β5i/LMP7^+/+^ and β5i/LMP7^-/-^ mice at d4 and d8 p.i. (n = 6 mice, representative for 3 independent experiments). (B) Cell numbers of B cells (CD19^+^, B220^+^) are illustrated as % of each cell population in comparison to the total number of cells from each spleen (left panel). CVB3-specific IgG levels were determined in sera collected at indicated time points, and titers of virus-specific IgG antibodies were determined by a CVB3-specific ELISA (n≥8 mice, right panel). (C) Ratios of CD4^+^ T cells (CD4^+^, CD3^+^) and CD8^+^ T cells (CD8^+^, CD3^+^) are illustrated. (D) For T cell transfer studies splenic CD8 T cells were separated by MACS (Miltenyi Biotec) from 1×10^7^ splenocytes (Fig. S2B). Left panel: To assess T cell survival after adoptive transfer, CD8 T cells were transferred from CVB3-infected β5i/LMP7^+/+^ (CD45.1) into β5i/LMP7^-/-^ (CD45.2) mice and from β5i/LMP7^-/-^ mice (CD45.2) into β5i/LMP7^+/+^ mice (CD45.1). Recipient mice were infected with CVB3 and sacrificed at d8 p.i. T cell survival was determined in splenocytes by flow cytometry. Middle panel: Prior to adoptive T cell transfer, β5i/LMP7^+/+^ and β5i/LMP7^-/-^ mice were infected with CVB3 and myocarditis scores were determined at d8 p.i. (n = 5 mice). Right panel: Then, splenic CD8 T cells were isolated from these CVB3-infected mice at d8 p.i. as described above; 1–2×10^6^ CD8^+^ T cells from one donor were injected i.v. through the tail vein into one recipient. Recipient mice were then infected with CVB3 i.p. and sacrificed at d8 p.i. Myocardial damage was evaluated in HE staining as described above. Data shown are mean for n = 5 mice and are representative for two independent experiments.

In line with the finding of identical viral load in CVB3-challenged cardiomyocytes from β5i/LMP7^+/+^ and β5i/LMP7^-/-^ mice, efficient virus elimination was observed at the chronic stage of disease at d28 p.i. revealing no relevant signs of ongoing disease in both hosts (data not shown). These findings pointed towards efficient induction of both innate and adaptive immunity also in mice lacking IP. To address this issue in detail, cytokine responses were determined in acute heart muscle injury (Fig. 5A). Our data demonstrate increased cardiac expression of pro-inflammatory cytokines as shown here exemplarily for TNF-α, IFN-β, IL-6 and IFN-γ in acute myocarditis in both β5i/LMP7^+/+^ and β5i/LMP7^-/-^ mice. Also, the expression of type I IFN-induced antiviral pathways like the 2′ 5′-oligoadenylate synthetase-like protein-2 (OASL-2), the IFN-stimulated gene 15 (ISG15), the Myxovirus resistance protein (Mx) and the protein kinase K (PKR) pathway was efficiently induced in CVB3-challenged hearts from β5i/LMP7^-/-^ mice (Fig. 5A).

To determine whether IP expression is required for maintaining homeostatic levels of B and T cells, we counted B220^+^/CD19^+^ B cells and CD4^+^ and CD8^+^ T cells in spleens isolated from β5i/LMP7^+/+^ and β5i/LMP7^-/-^ mice. Hensley et al. recently reported on different absolute B cell and T cell numbers and impairment in humoral immunity in β1i/LMP2-deficient mice [30]. Here, we detected equal CD4^+^ T cell and B cell numbers in both CVB3-challenged β5i/LMP7^+/+^ and β5i/LMP7^-/-^ mice (Fig. 5B, C). To further test humoral immunity in β5i/LMP7^-/-^ mice, anti CVB3 IgG titers were determined at different time points p.i. CVB3-antibody titers in β5i/LMP7^-/-^ mice were found to be within the range of anti CVB3 IgG in respective wildtype controls (Fig. 5B). In accordance with data by Fehling et al. [21], CD4^+^ and CD8^+^ T cells were not affected by β5i/LMP7 deletion in naive mice (Fig. 5C). We observed an overall decrease in splenic T cell levels early upon disease potentially reflecting migration of these cells to secondary lymphoid organs and to the target organ of infection; however, host-specific effects were precluded. Also, we did not observe any differences in the absolute number of splenocytes in both hosts in acute myocarditis.

Since β5i/LMP7-deficiency has been attributed to impaired CD8 T cell responses [21], [31], we also investigated CD8 T cell populations from both β5i/LMP7^+/+^ and β5i/LMP7^-/-^ mice *in vivo*. Despite the fact that absolute frequencies of CVB3 VP2 [285–293]-specific CD8 T cells were rather low, no remarkable differences were detected in the absolute VP2 [285–293]-peptide specific CD8 T cell numbers in CVB3-infected β5i/LMP7^+/+^ and β5i/LMP7^-/-^ mice (Fig. S2A). Given that CD8 T cells are crucial in the control of CVB3-myocarditis [29], [32], absolute CD8 T cell numbers for CVB3 epitopes are low and detection of specific CD8 T cell frequencies is limited in this infection model, adoptive transfer studies of CVB3-memory CD8 T cells from IP-deficient and IP-competent mice appeared to be the most reliable approach to address the effect of IP-deficiency on CD8 T cell function. Take of donor CD8 T cells from CVB3-infected mice is shown in Fig. S2B. To preclude effects of IP-deficiency on CD8 T cell survival, CD8 T cells were isolated from CVB3-challenged β5i/LMP7^+/+^ and β5i/LMP7^-/-^ mice (both CD45.2) at d8 p.i. and transferred into naive B6.SJL-Ptprca Pepcb/BoyJ mice (CD45.1). The amount of transferred CD8 T cells was assessed at d8 after adoptive T cell transfer revealing no impairment of IP-deficiency on CD8 T cell survival (data not shown). Also, transfer of β5i/LMP7-deficient CD8 T cells (CD45.2) into CVB3-infected B6.SJL-Ptprca Pepcb/BoyJ mice (CD45.1) and vice versa revealed comparable CD8 T cell survival rates (Fig. 5D left panel). Next, CD8 T cells were isolated from CVB3-infected β5i/LMP7^+/+^ and β5i/LMP7^-/-^ mice (both CD45.2) at d8 p.i. These cells were transferred into naive β5i/LMP7^+/+^ and β5i/LMP7^-/-^ mice, which were then infected with CVB3 and sacrificed at d8 p.i. to assess myocarditis scores. Following adoptive T cell transfer of IP-deficient CD8 T cells into either IP-deficient or IP-competent recipients, we observed no effect on acute heart muscle inflammation in comparison to adoptive T cell transfer of CD8 T cells from IP-competent mice into both recipients (Fig. 5D right panel). Of note, adoptive T cell transfer of CD8 T cells from either CVB3-infected β5i/LMP7^+/+^ and β5i/LMP7^-/-^ mice into β5i/LMP7^-/-^ recipient mice resulted in a slightly milder acute disease than in non-transfected mice (respective myocarditis score from CVB3-infected donor mice are shown in the middle panel of Fig. 5D). However, these effects were detected for T cell transfer of both β5i/LMP7^-/-^ and β5i/LMP7^+/+^ T cells, thus arguing against a detrimental effect of IP-deficiency on memory CD8 T cell function and being in accordance with the observation of equal virus titers and efficient viral clearance in both β5i/LMP7^+/+^ and β5i/LMP7^-/-^ mice.

### IP-deficient hearts are prone to oxidant protein damage and accumulation of poly-ub conjugates

**Figure 6:**
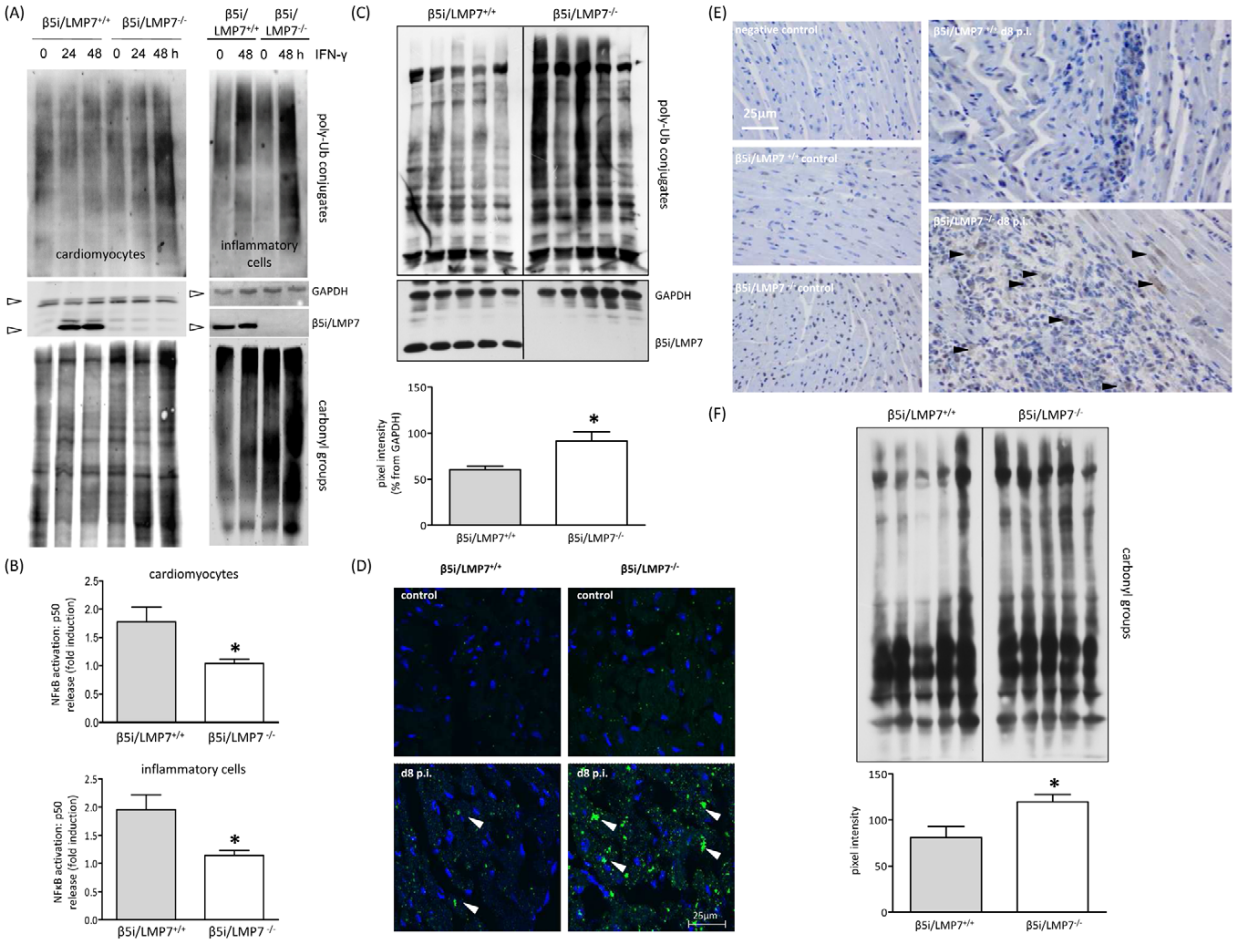
Accumulation of poly-ub protein conjugates in IP-deficient mice. (A) Primary cardiomyocytes and B-cell depleted splenocytes from β5i/LMP7^+/+^ and β5i/LMP7^-/-^ mice were incubated with IFN-γ for indicated time points. Lack of IP formation resulted in accumulation of poly-ub substrates and of oxidant-damaged proteins (staining of carbonyl groups) upon IFN-γ exposure in both cell populations (cell purity is depicted in Fig. S1). GAPDH illustrates equal protein loading. Images are representative from at least two independent experiments. (B) NFκB p50 levels were determined in whole cell homogenates from IFN-γ (100 U/ml for 24 h) treated primary cardiomyocytes and B-cell depleted splenocytes upon 30 min TNF-α exposure. Data are shown as fold increase of p50 levels in comparison to untreated control cells (representative mean from triplicates from at least two independent experiments). (C) Immunoblot of CVB3-infected cardiac homogenates from five individual β5i/LMP7^+/+^ and β5i/LMP7^-/-^ mice (d8 p.i.) stained for poly-ub-conjugates (from the same transfer; representative for n = 10 mice). Densitometry was performed for individual lanes for these five different mice (normalization to GAPDH control) yielding increased levels of poly-ub signals in β5i/LMP7^-/-^ mice (mean±SEM). (D) Formation of poly-ub containing ALIS (white arrowheads) was visualized by immunofluorescence: heart cryosections were stained with FK1 for poly-ub (green) and Hoechst (blue). Here, slides are representative for n = 6 mice from two independent experiments (more data is illustrated in Fig. S3). (E) To localize poly-ub conjugates within the injured myocardium, immunohistology staining of ubiquitin was performed in control and CVB3-infected mice (d8 p.i.). Representative tissue sections are shown. For more information see Fig. S4 to this figure. (F) CVB3-challenged cardiac lysates from from five individual β5i/LMP7^+/+^ and β5i/LMP7^-/-^ mice (d8 p.i.; representative for n = 9 mice) stained for oxidant-damaged proteins by staining of carbonyl groups. Quantitative evaluation of oxy-staining was performed as described above. All statistical data are mean±SEM; * p<0.05 as determined by Mann-Whitney U test.

The data above illustrated severe tissue damage in CVB3-challenged hearts in mice lacking IPs and revealed large foci of inflammatory lesions in this host. Given that IPs preserve protein homeostasis and cell viability in response to cytokine stress [24], one may argue that viral infection induced cytokine release affects the cellular protein equilibrium in cardiomyocytes and invading inflammatory cells, which may further exacerbate heart muscle injury in IP-deficient hearts. To test this hypothesis, primary cardiomyocytes and B-cell depleted splenocytes (which represent the major populations of invading inflammatory cells) were isolated from β5i/LMP7^+/+^ and β5i/LMP7^-/-^ mice and exposed to IFN-γ (cell purity is depicted in Fig. S1). Upon cytokine exposure, lack of IPs resulted in increased accumulation of poly-ub substrates in these cells (Fig. 6A). Failure of IP expression also coincided with increased accumulation of oxidant-damaged proteins in IP-deficient cardiomyocytes and inflammatory cells in response to prolonged cytokine exposure (Fig. 6A). IPs also contribute to the activation of NFκB transcription factor by accelerated turnover of IκBα, which is crucial for multiple processes in inflammation and apoptosis [24], [33]. Impaired activation of NFκB as shown here by reduced levels of NFκB p50 subunits in IP-deficient cardiomyocytes and inflammatory cells (Fig. 6B) reflected reduced proteasomal degradation of NFκB p105 precursor proteins, which is in concordance with impaired proteolysis in IP-deficient cells as shown in Fig. 6A.

These data suggested a role of IPs in regulating proteotoxic stress also in the infected myocardium. Indeed, IP-deficient mice failed to cope with accelerated protein turnover in CVB3 infection as reflected by increased accumulation of poly-ub proteins in acute inflammatory viral heart disease (Fig. 6C). The IP-deficient myocardium was not able to efficiently cope with the required protein turnover in acute CVB3 myocarditis (Fig. 6D + Fig. S3). Consequently, we observed significantly enhanced ALIS formation in the injured myocardium (evaluation of poly-ub-aggregates at the acute stage of myocarditis: β5i/LMP7^+/+^ mice: 13.5 ALIS / 1088 µm^2^±1.0 vs. β5i/LMP7^-/-^ mice: 20.0 ALIS / 1088 µm^2^±1.8; p<0.05; n = 5 mice). These poly-ubiquitin conjugates were primarily detected within inflammatory lesions in invading inflammatory cells, and within the cytoplasm and nuclei of adjacent cardiomyocytes in acute myocarditis (Fig. 6E). Also, poly-ub signals were found to be increased in β5i/LMP7^-/-^ mice in comparison to β5i/LMP7^+/+^ mice at d8 p.i. (Fig. 6E + Fig. S4). Since oxidant damaged proteins become substrates of the 26S IP upon tagging by poly-ub [24], the levels of carbonyl groups reflecting oxidant protein damage were monitored. As illustrated in Fig. 6F, oxidant protein damage was increased in acutely inflamed hearts in IP-deficient mice.

### IP-deficient hearts are prone to apoptotic cell death

**Figure 7:**
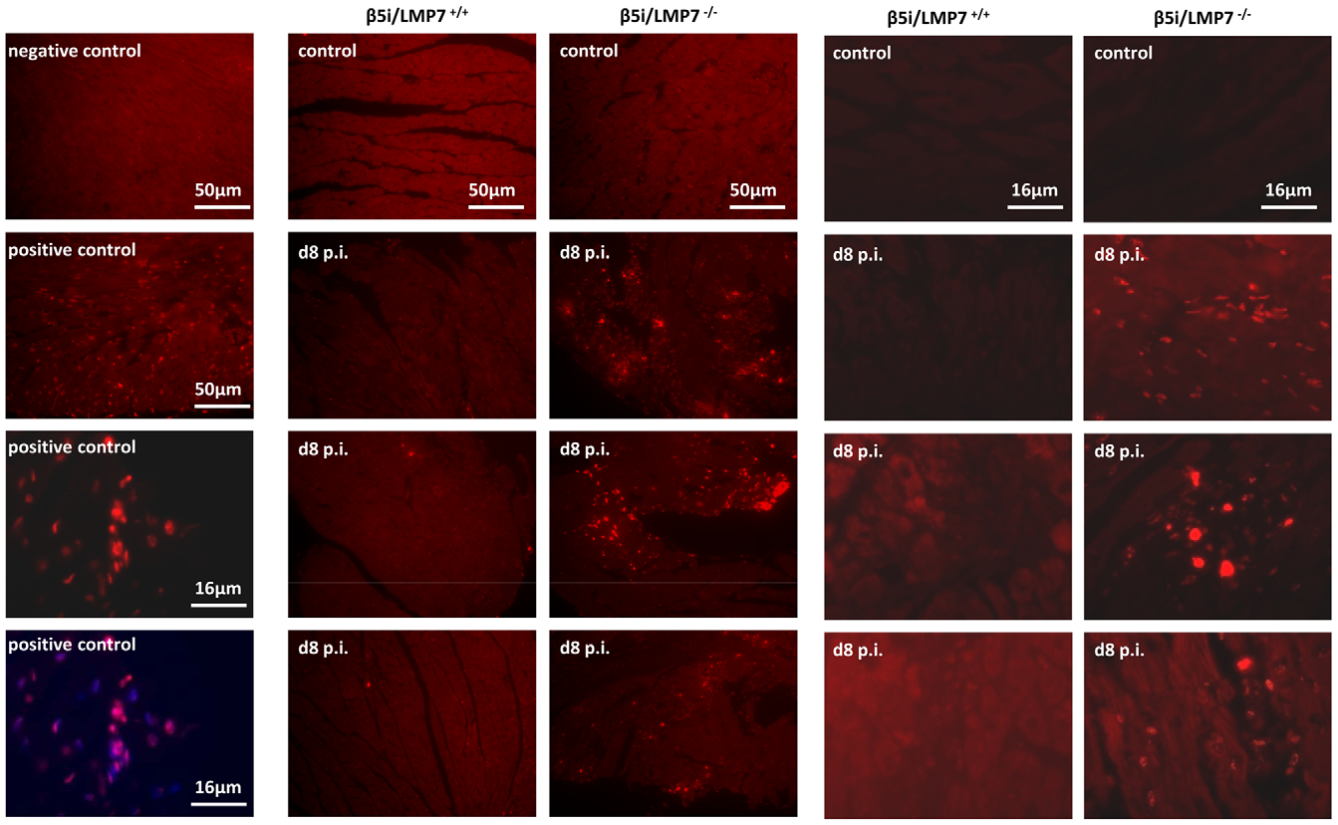
IP-deficient hearts are prone to apoptotic cell death. Apoptosis was assessed *in vivo* in cardiac tissue sections in naive and CVB3-challenged mice (d8 p.i.) by *in situ* cell death detection kit, TMR red. Representative cardiac sections from two independent experiments (infection of n = 5 mice / experiment) are shown. Left panels: controls - upper two slides from first experiment, lower two slides with positive controls from second experiment illustrating DNA strand breaks in inflammatory lesions and surrounding cardiomyocytes, which colocalize to nuclei (merge with Hoechst staining). More information is provided in Fig. S5 + 6 to this figure.

Since CVB3 titers were found to be within the same range in both hosts (Fig. 4), cytolytic effects of CVB3 do apparently not explain severe tissue injury as observed here in IP-deficient mice. However, oxidative-protein damage, inefficient degradation of poly-ub protein aggregates and reduced activation of NFκB transcription factor in CVB3-challenged hearts in mice lacking IPs may affect cell viability. Indeed, cytokine-induced cellular injury predominantly occurred *in vitro* in cardiomyocytes and macrophages that were isolated from IP-deficient mice (data not shown). To study cellular injury due to apoptotic cell death *in vivo*, DNA strand breaks as an early sign of apoptosis were assessed in cardiac tissue sections. No apoptotic cell death was detected in hearts from β5i/LMP7^+/+^ and β5i/LMP7^-/-^ mice at d4 p.i. (Fig. S5). However, in acute heart muscle injury (d8 p.i.), increased levels of DNA strand breaks were detected particularly within inflammatory lesions and the surrounding tissue in CVB3-challenged β5i/LMP7^-/-^ mice (Fig. 7, Fig. S5+S6). TUNEL positive staining was detected throughout the injured heart in IP-deficient mice; thus, apoptotic cell death was found to be quantitatively increased in β5i/LMP7^-/-^ mice. Despite the fact that minor inflammatory lesions were also detected in CVB3-infected β5i/LMP7^+/+^ mice (d8 p.i.), here no significant apoptotic cell death occurred (Fig. 7). This observation is in agreement with previously published data [29]. These findings support the role of IP formation in cardiomyocytes and in inflammatory cells to protect the injured tissue from proteotoxic stress, which may exacerbate acute heart muscle injury in viral heart disease.

## Discussion

Proteasomes are responsible for the generation of peptides derived from pathogens or cellular proteins that are presented by MHC class I molecules on the cell surface to cytotoxic T cells (CTLs) [11]. Despite the fact that *in vitro* studies argued in favor of an impact of IP-dependent MHC class I antigen processing [15]–[17], [19], *in vivo* studies using IP-deficient mice reported conflicting data on the induction of CD8 T cell responses [17], [21], [23], [34]. CD8 T cells are crucial in virus elimination in CVB3-myocarditis [29], [32]. To study a potential contribution of IPs to the generation of CD8 T cell responses in CVB3-myocarditis, here adoptive memory CD8 T cell transfer experiments were performed since limited knowledge on immunodominant CVB3-specific CD8 T cell epitopes restrains solid quantification of CD8 T cell responses in murine enterovirus myocarditis [19], [35]. Transfer of CVB3 memory CD8 T cells from IP-competent mice did not reveal a beneficial effect on CVB3 myocarditis in comparison to transfer of CD8 T cells from IP-deficient mice. Likewise, CVB3 titers were within the same range in IP-deficient and wildtype control mice in acute myocarditis (Fig. 4) and the virus was efficiently eliminated in both hosts at d28 p.i. These findings support the induction of efficient CD8 T cell responses also in CVB3-challenged IP-deficient mice, which is in agreement with observations in other infection models: the kinetics of lymphocytic choriomeningitis virus clearance were similar in both β5i/LMP7^+/+^ and β5i/LMP7^-/-^ mice [34]. This strongly supports the notion that the key innate function of IP in enteroviral heart disease lies elsewhere. Here, we have illustrated that IPs in CVB3-induced heart muscle injury preserve protein homeostasis and maintain cell viability in order to protect the inflammation-challenged myocardium from severe damage.

The UPS adapts to stress induced requirements by increased substrate turnover exerted by IPs, which possess improved peptide-hydrolyzing activity [15], [17], [35], and poly-Ub-substrate turnover [24]. Indeed, cardiac IP formation in CVB3-myocarditis resulted in enhanced proteasomal peptide-hydrolyzing activity [19]. One of the pivotal functions of the UPS is to limit the accumulation of potentially toxic misfolded proteins and protein aggregate formation, which as consequence of cellular stress represent a constant threat to normal cell function and cell viability [7], [36]. Also, CVB3-infection [2] as well as cytokine stress in inflammation [1] induce oxidative stress. Likewise, the pathogenesis of severe CVB3 myocarditis has been attributed to increased oxidative stress [37]–[39]. In agreement with our previous study reporting on the activity of the 26S IP for efficient elimination of oxidatively modified, poly-ub proteins in response to cytokine stress [24], here we demonstrated that IP function in both residing host cells and invading inflammatory cells is crucial for the efficient degradation of poly-ub proteins in acute viral heart disease. Elimination of nascent oxidant-damaged, poly-ub proteins by the 26S IP prevented the accumulation of harmful protein aggregates [7], [36], which may exacerbate acute heart muscle injury.

Moreover, as a consequence of impaired IP function, poly-ub proteins accumulated in ALIS in CVB3-infected hearts. Such aggregates, which are at least partially comprised of CVB3 proteins [40], are not inert metabolic end products, but may actively influence the metabolism of cells [41]. As shown here, degradation of oxidant-damaged, poly-ub proteins by cardiac 26S IPs in CVB3-challenged hearts resulted in ALIS degradation and likewise protected cardiomyocytes and invading inflammatory cells from proteotoxic stress. This first histological demonstration of severe tissue injury in virus infection in mice lacking IPs is in agreement with findings in experimental acute encephalomyelitis (EAE). IPs prevented accumulation of toxic protein aggregates in EAE, coinciding with less severe disease manifestation in IP-competent mice [24]. The detection of both poly-ub proteins in concert with apoptotic cell injury within inflammatory lesions in viral heart disease (Fig. 6+7) also supports the association between proteotoxic stress and cellular injury in this model [7], [24]. In fact, cellular injury as shown here by increased apoptotic cell death of invading inflammatory cells and adjacent cardiomyocytes in IP-deficient mice may result in the release of endogenous molecules, which as damage-associated molecular patterns (DAMPs) signal the threat of infection and injury to the organism. High levels of DAMPs have been linked to the pathogenesis of many inflammatory diseases, drive cellular activation and immunoreactivity [42]. This may in fact exacerbate acute inflammation and also result in killing of non-infected cardiomyocytes. Thus, IPs prevent excessive proteotoxic stress and cellular injury, which in consequence may limit additional effects like DAMP-associated activation of immunopathology.

Remarkably, this is the first study illustrating a detrimental effect of IP-deficiency in a viral infection-induced phenotype despite a lack of a significant effect of IPs on pathogen load. Our findings are in agreement with the observation that absence of β5i/LMP7 expression impairs the beneficial effects of IFN-β in patients suffering from multiple sclerosis [43]. In contrast, absence of IP-function either as a result of β5i/LMP7 deficiency or inhibition of β5i/LMP7 catalytic activity by PR-957 have recently been associated with attenuated experimental colitis [44]. Depending on the pathogenesis of the underlying disease, IP deficiency seems to exert either protective effects or to aggravate the consequences of inflammation in a disease or tissue-specific manner. Indeed, cytokine responses in IP-deficient mice differ considerably and are strikingly dependent on the disease entity being studied. CVB3 infection of IP-deficient mice revealed a cytokine induction in viral heart muscle injury comparable to that observed in wildtype mice (Fig. 5A). In contrast, amelioration of experimental colitis has been connected to limited induction of proinflammatory cytokines and chemokines [33], [44]. This was attributed to impaired activation of transcription factor NFκB, a central regulator of inflammation in inflammatory bowel disease [33]. In line with impaired NFκB activation in TNF-α stimulated murine embryonic fibroblasts [33], this study demonstrated reduced NFκB activation in cytokine-stressed cardiomyocytes and inflammatory cells lacking IPs (Fig. 6B). This may be attributed to the fact that IκBα, a specific inhibitor of NFκB activation, is degraded much faster in cells expressing IPs [45]. Also, IκBα has been identified to be oxidatively-modified upon cytokine stress, which supports the role of IPs in degradation of this specific substrate [24]. Whereas increased activation of NFκB is believed to exert detrimental functions in immune and non-immune cells in tissues affected by chronic inflammation, NFκB inhibition can also be harmful for the organism, and trigger the development of inflammation and disease. These findings suggested that NFκB signaling has important functions for the maintenance of physiological immune homeostasis and for the prevention of inflammatory diseases [46]. Studies with specific inhibitors of NFκB nuclear translocation and activity revealed induction of apoptosis, thus argueing in favor for anti-apoptotic effects of this prosurvival transcription factor as well [47]. In agreement with these findings, the here described impaired activation of NFκB may additionally contribute to the effects of proteotoxic stress, which resulted in cellular injury as shown in Fig. 7. Whereas interference with two major pathways leading to NFκB activation exerts beneficial effects in experimental colitis and anti-cancer treatment, our data indicate that activation of NFκB-mediated responses protects cytokine-challenged cardiomyocytes and inflammatory cells and argues against a significant contribution of NFκB to cytokine induction in viral heart disease. In conclusion, our findings support the view of a distinct tissue specific contribution of IP function driven by the pathogenesis of the underlying inflammatory disease.

## Material and Methods

### Ethics statement

This study was carried out in strict accordance with the recommendations in the Guide for the Care and Use of Laboratory Animals of the German animal welfare act, which is based on the directive of the European parliament and of the council on the protection of animals used for scientific purposes. This study conforms to the Berlin State guidelines for animal welfare. The protocol was approved by the Committee on the Ethics of Animal Experiments of Berlin State authorities (Permit Number: G0311/06). All efforts were made to minimize suffering.

### Mice

CVB3 (cardiotropic Nancy strain) used in this study was prepared as previously described [48]. C57BL/6 mice were initially from Jackson Laboratory. C57BL/6 β5i/LMP7^-/-^ mice originally obtained from HJ Fehling [21], who backcrossed them with C57BL/6 mice 10 times. Breeding pairs were kindly provided by U Steinhoff (Berlin, Germany). Mice were kept at the animal facilities of the Charité University Medical Center. Six week-old mice were infected i.p. with 1×10^5^ PFU CVB3. Hearts were perfused with PBS, weighted and quickly frozen in liquid nitrogen before storage at -80°C. For some lymphocyte transfer experiments, B6.SJL-Ptprca Pepcb/BoyJ were purchased from Jackson Laboratory.

### Cell culture

Primary cardiomyocytes (CM) were isolated from fetal mouse hearts (E13). Hearts were incubated in EDTA/Trypsin at 4°C overnight, followed by 15 min incubation at 37°C. Cardiac cells were resuspended in standard medium and transferred into cell culture flasks. Purity was determined by flow cytometry using cardiomyocyte-specific troponin I antibodies (abcam # 47003). Inflammatory cells were taken from whole spleen cell suspensions, which were B cell depleted by MACS (Miltenyi Biotec). All cell lines were cultivated under standard conditions in Dulbecco's MEM (MEFs) each containing 10% fetal calf serum (FCS), 2 mM L-glutamine, 100 U/ml penicillin and 100 mg/ml streptomycin. Cells were treated with either 100 U/ml IFN-β, 100 U/ml IFN-γ (Sigma-Aldrich) or 30 ng/ml TNF-α (all Sigma-Aldrich). Cells were infected with CVB3 (cardiotropic Nancy strain) MOI 0.1 or 0.5 as indicated. Plaque assay was performed as described previously [19].

### In situ hybridization and histological staining

*In situ* hybridization of genomic CVB3 RNA, histological staining with hematoxylin / eosin (HE) and immunohistochemistry for detection of CD3^+^ T lymphocytes and Mac-3^+^ macrophages were carried out and analysed as described [27]. Immunohistochemical stainings for ubiquitin and B cells were performed on a Ventana Benchmark stainer using the Vectostain Elite ABC Kits (Vector Laboratories; Burlingame, CA). The following primary antibodies were used: anti-ubiquitin 1∶1000 (DAKO Cytomation, Glostrup, Denmark) and CD45R/B220 1∶100 (BD Pharmingen, Heidelberg, Germany). Biotin-labeled secondary antibodies (goat-anti-rabbit and goat-anti-rat) were purchased from Jackson Immuno Research (Dianova, Hamburg, Germany) and used at a dilution of 1∶100. All slides were counterstained with hematoxylin.

### Immunofluorescence

Processing of cryo-sections and Hoechst staining was performed as described [24]. Briefly, cryo-sections were fixed in 4% paraformaldehyde, washed and permeabilized with PBS/1% TritonX. Staining was performed with FK1 mAb (PW 8805, Biomol, Germany) at 4°C over night. Confocal images were acquired on a Leica TCS SP2 microscope (Leica Microsystems). Quantification of ALIS has been based on counting cells with accumulation of poly-ub conjugates (focused staining over background defined as ub-rich inclusions) in defined areas (1088 µm^2^) at 100-fold magnification.

### TaqMan quantitative RT-PCR

RNA preparation and cDNA synthesis were performed as described recently [19]. TaqMan PCR was performed using primers and probes of TaqMan Gene Expression Assays (Applied Biosystems, Germany). mRNA expression was normalized to the housekeeping gene HPRT by means of the ΔCt method.

### Immunoblot analysis

Cell or tissue lyses was performed in 20 mM TRIS-HCl, pH 7.5, 10 mM EDTA, 100 mM NaCl, 1% NP40, 10 µM MG132, 5 mM NEM, Complete protease inhibitor cocktail (Roche, Germany). Immunoblot analysis was performed according to standard protocols. ubiquitin: Z0458 DAKO; α6 (pc, K379), β5i/LMP7 (pc, K63), β2i/MECL-1 (pc, K65): lab stock (all generated against peptides of the respective protein), β1i/LMP2: Abcam #3328 for isolated proteasomes or β1i/LMP2: lab stock for cardiomyocytes (cross-reaction with β1, pc, K620/21); α-actin, GAPDH: Santa Cruz.

### Determination of oxidative stress and apoptosis

The detection of oxidatively-damaged proteins was performed indirectly by chemical derivatization: this derivatization captures the oxidative state immediately during or after homogenization of the tissue. Oxidized proteins were visualized with the OxyBlot protein oxidation detection kit (Chemicon International) via immunodetection of carbonyl groups. DNA strand breaks (TUNEL assay) were determined by *in situ* cell death detection kit, TMR red (Roche, Germany) or *in situ* cell death detection kit, POD (Roche, Germany) according to the instructions of the manufacturer. POD stained slides were counterstained by hematoxylin.

### NFκB ELISA

After induction of IPs with IFN-γ, primary cardiomyocytes and B-cell depleted splenocytes were stimulated with 30 ng/ml TNF-α for 30 min. p50 NFκB was determined in whole tissue homogenates by ELISA according to the manufacture's instructions (ActiveMotif, Rixensart, Belgium).

### Detection of CVB3-specific antibodies by ELISA

CVB3-specific IgG antibody titers were determined with Enterovirus ELISA Kit (Genzyme Diagnostics) according to the manufacture's instructions [alternative secondary antibody (POX anti-mouse IgG, Dianova) was used]. CVB3-specific antibody titers are presented as log_2_ of the maximum dilution of serum showing an optical density greater than the mean optical density of sera obtained from naive mice plus threefold SD as described recently [27].

### Flow cytometry

After Fc-receptor blockade cells were incubated with different combinations of fluorescently labeled Abs (eBiosciences and BD Biosciences) and samples were analyzed using CYAN-ADP flow cytometer (Beckman Coulter, Germany) or BD FACSCalibur (Becton Dickinson).

### Lymphocyte transfer

At day 8 p.i., CVB3-infected mice were sacrificed and splenocytes were isolated according to standard protocols. CD8^+^ T cells were purified by positive selection using commercially available kits yielding a purity of at least 85% (Miltenyi Biotec). 1-2×10^6^ CD8^+^ T cells from one donor were injected i.v. through the tail vein into one recipient. After T cell transfer, mice were injected i.p. with 1×10^5^ PFU CVB3 and sacrificed at the acute stage of infection at d8 p.i. Myocarditis was assessed as described above.

### Quantification of proteasome subunits

20S proteasomes were isolated according to standard procedure as described [19]. The mixture of tryptic peptides was separated prior to mass spectrometric analyses by reverse phase nano HPLC on a 15 cm PepMap100-column (3 µl, 100 Å) using an Proxeon System (Odense, Denmark) at a flow rate of 1 µl/min. Separation was carried out in a linear gradient within 86 min using 0.05% acetic acid, 2% acetonitrile in water and 0.05% acetic acid in 45% acetonitrile as eluents. MS-data were generated on an LTQ-Orbitrap-MS equipped with a nanoelectrospray ion source (PicoTip Emitter FS360-20-20-CE-20-C12, New Objective). After a first survey scan (r = 60,000) MS2 data were recorded for the five highest mass peaks in the linear ion trap at a collision induced energy of 35%. The exclusion time was set to 30 s and the minimal signal for MS2 was 1,000. Peptide identification was achieved by searching the SwissProt database rel. 57.1 restricted to mouse entries using SEQUEST search engine (SageN Research) and further processed by PeptideTeller and ProteinTeller [49] within the Elucidator system (Rosetta Biosoftware, Seattle, WA, U.S.A.). ProteinTeller results were further used for annotation, with a predicted error rate of<5%. Quantitative analysis of label-free MS data was achieved with the Elucidator system using peptide intensities as proxies for label-free peptide abundance measurements. The following criteria for frame and feature annotation were used: retention time minimum cut-off 9 min, retention time maximum cut-off 80 min, m/z minimum cut-off 300, instrument mass accuracy 5 ppm, alignment search distance 10 min. For quantitative analysis, the data were normalized and further grouped (two biological and two technical replicates).

### Statistics

Results of continuous variables are expressed as mean±standard error of mean (SEM) if not indicated otherwise. Two group comparisons of non-parametric data were performed using the Mann-Whitney test. Statistical significance between multiple groups was determined using two-way ANOVA and post hoc analysis with a Bonferroni test. Significance was assessed at the p<0.05 level (* indicates significant differences).

### Accession numbers

TNF-α (Q0X0E6, P06804), IFN-β (P01575), IL-6 (P08505), IFN-γ (P01580), OASL-2 (Q9Z2F2), ISG15 (Q64339), Mx (P09922), PKR (Q03963), NFκB p105 (P25799), IκBα (Q9Z1E3), TLR7 (P58681), TLR8 (P58682), LMP2 (P28076), MECL-1 (O35955), LMP7 (P28063), CD8 (P01731), CD68 (P31996), CD3 (P22646), B220 (P06800), CD4 (P06332), CD19 (P25918)

## Supporting Information

Figure S1 **Characterization of primary cardiomyocytes and inflammatory cells.** (A) Primary cardiomyocytes (CM) were isolated from fetal mouse hearts. Purity was determined by flow cytometry using cardiomyocyte-specific troponin I antibodies revealing >93% troponin I^+^ cells. Representative histograms are shown (unstained cells in upper panels, troponin I / Alexa488-anti rabbit IgG staining in lower panels). Also, after cell adherence all visible cells demonstrated spontaneous contraction in cell culture, thus representing a common hallmark of cardiomyocytes in addition to specific troponin I expression. (B) Inflammatory cells: representative histograms illustrate B220^+^/CD19^+^ B cells from whole spleen cell suspensions prior to and after B cell depletion by MACS.
(PDF)

Figure S2 **Viral epitope-specific T cell responses in acute myocarditis and take of CD8 donor T cell for adoptive transfer experiments.** (A) Pentamer staining of VP2 [285–293]-specific CD8 T cells. H-2^b^- PE pentamers for virus capsid protein 2 (VP2) [285–293] was purchased from ProImmune (Oxford, UK). Splenic CD8 T cells from naive and CVB3-infected β5i/LMP7^+/+^ and β5i/LMP7^-/-^ mice (d8 p.i., n = 10) were stained according to the instructions of the manufacturer (Pentamer-PE, CD8-FITC, CD19-PE Cy5) as described recently [19]. Pentamer-positive VP2 [285-293]-specific CD8 T cell numbers (1×10e5 gated cells) did not differ between the two hosts. (B) For T cell transfer studies splenic CD8 T cells were separated by MACS (Miltenyi Biotec) from 1×10^7^ splenocytes (splenocytes taken from CVB3-infected mice). Purity of respective CD8 T cell populations for T cell transfer studies was >85%. Representative dot plots indicate the amounts of CD8^+^/CD3^+^ T cells post-MACS.
(PDF)

Figure S3 (to Fig. 6D) **Accumulation of poly-ub protein conjugates in IP-deficient mice.** Formation of ALIS was visualized by immunofluorescence. Heart cryosections from naive and CVB3-infected β5i/LMP7^+/+^ and β5i/LMP7^-/-^ mice (d8 p.i.) were stained with FK1 for poly-ub (green). Slides from four different individual mice are shown (representative for n = 6 mice from two independent experiments).
(PDF)

Figure S4 (to Fig. 6E) **Accumulation of poly-ub protein conjugates in IP-deficient mice.** To visualize poly-ubiquitin conjugates within the injured myocardium, immunohistology staining of ubiquitin was performed in control and CVB3-infected mice (sacrificed at d4 and d8 p.i.). 1^st^ column controls: negative controls reflect secondary antibody staining only within the myocardium (upper panel) and within an inflammatory focus (lower panel). Ubiquitin staining is shown for two representative naive mice from β5i/LMP7^+/+^ and β5i/LMP7^-/-^ mice. 2^nd^ column d4 p.i. Ubiquitin staining is shown for three different mice from each host. No specific increase in ubiquitin signals is visualizable at this time point. 3^rd^–5^th^ column d8 p.i. Ubiquitin staining is illustrated for three different mice representing at least n = 5 mice / host. Three different tissue sections are shown per mouse indicating increased poly-ub detection in CVB3-infected β5i/LMP7^-/-^ mice.
(PDF)

Figure S5 (to Fig. 7) **IP-deficient hearts are prone to apoptotic cell death.** Apoptosis was assessed *in vivo* in cardiac tissue sections in naive and CVB3-challenged mice (d4 and d8 p.i.) by *in situ* cell death detection kit, TMR red. Representative cardiac sections from one independent experiment are shown. (A) Controls: lower two slides illustrate DNA strand breaks within inflammatory lesions and surrounding cardiomyocytes, which colocalize to nuclei (last slide). (B) TUNEL staining is shown for one control heart and four individual mice sacrificed at d4 p.i. (representative for n = 6 mice). No apoptotic cell death was detected in these mice. (C) DNA strand breaks were visualized in CVB3-infected hearts from β5i/LMP7^-/-^ mice: three representative slides are shown for each heart. Increased levels of TUNEL-positive cells are located particularly within inflammatory lesions and surrounding cardiomyocytes. In CVB3-infected β5i/LMP7^+/+^ mice hardly any apoptotic lesions were detected (1^st^ row representative for n = 8 mice).
(PDF)

Figure S6 (to Fig. 7) **Apoptotic cell death within inflammatory lesions in IP-deficient mice.** To further localize cellular injury within the injured myocardium, *in situ* cell death detection kit, POD was used. Tissue sections are representative for at least n = 5 mice.
(PDF)
