# Supplementary figures and images for: Impairment of Immunoproteasome Function by β5i/LMP7 Subunit Deficiency Results in Severe Enterovirus Myocarditis
Source: PLoS Pathog. 2011 Sep 1;7(9):e1002233. doi: 10.1371/journal.ppat.1002233 (PMC3164653; doi:10.1371/journal.ppat.1002233)

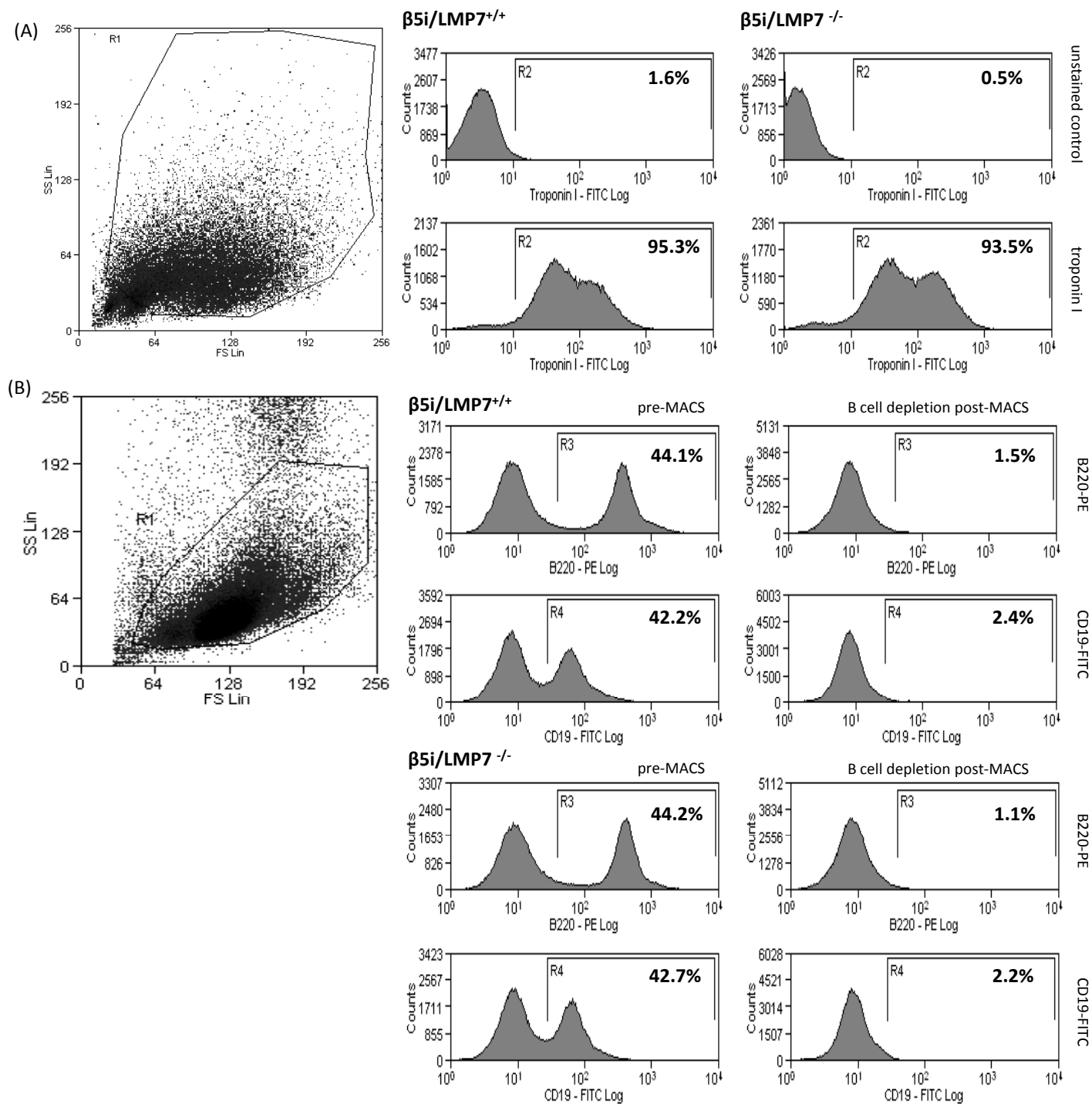

Supplement: Figure S1 — Characterization of primary cardiomyocytes and inflammatory cells. (A) Primary cardiomyocytes (CM) were isolated from fetal mouse hearts. Purity was determined by flow cytometry using cardiomyocyte-specific troponin I antibodies revealing >93% troponin I+ cells. Representative histograms are shown (unstained cells in upper panels, troponin I / Alexa488-anti rabbit IgG staining in lower panels). Also, after cell adherence all visible cells demonstrated spontaneous contraction in cell culture, thus representing a common hallmark of cardiomyocytes in addition to specific troponin I expression. (B) Inflammatory cells: representative histograms illustrate B220+/CD19+ B cells from whole spleen cell suspensions prior to and after B cell depletion by MACS. (PDF) [file ppat.1002233.s001.pdf]

(A)

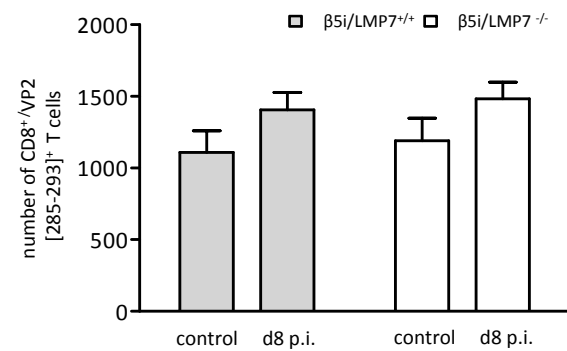

(B)

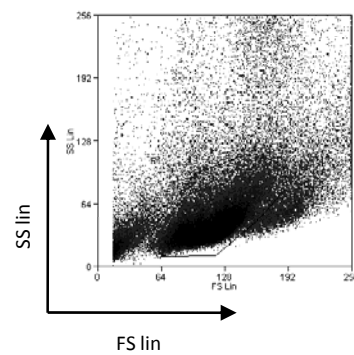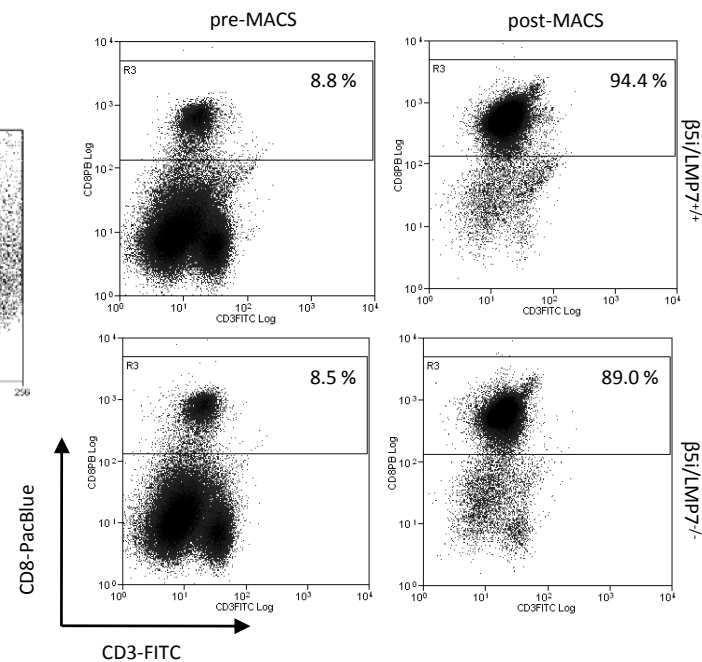

Supplement: Figure S2 — Viral epitope-specific T cell responses in acute myocarditis and take of CD8 donor T cell for adoptive transfer experiments. (A) Pentamer staining of VP2 [285–293]-specific CD8 T cells. H-2b- PE pentamers for virus capsid protein 2 (VP2) [285–293] was purchased from ProImmune (Oxford, UK). Splenic CD8 T cells from naive and CVB3-infected β5i/LMP7+/+ and β5i/LMP7-/- mice (d8 p.i., n = 10) were stained according to the instructions of the manufacturer (Pentamer-PE, CD8-FITC, CD19-PE Cy5) as described recently [19]. Pentamer-positive VP2 [285-293]-specific CD8 T cell numbers (1×10e5 gated cells) did not differ between the two hosts. (B) For T cell transfer studies splenic CD8 T cells were separated by MACS (Miltenyi Biotec) from 1×10^7^ splenocytes (splenocytes taken from CVB3-infected mice). Purity of respective CD8 T cell populations for T cell transfer studies was >85%. Representative dot plots indicate the amounts of CD8+/CD3+ T cells post-MACS. (PDF) [file ppat.1002233.s002.pdf]

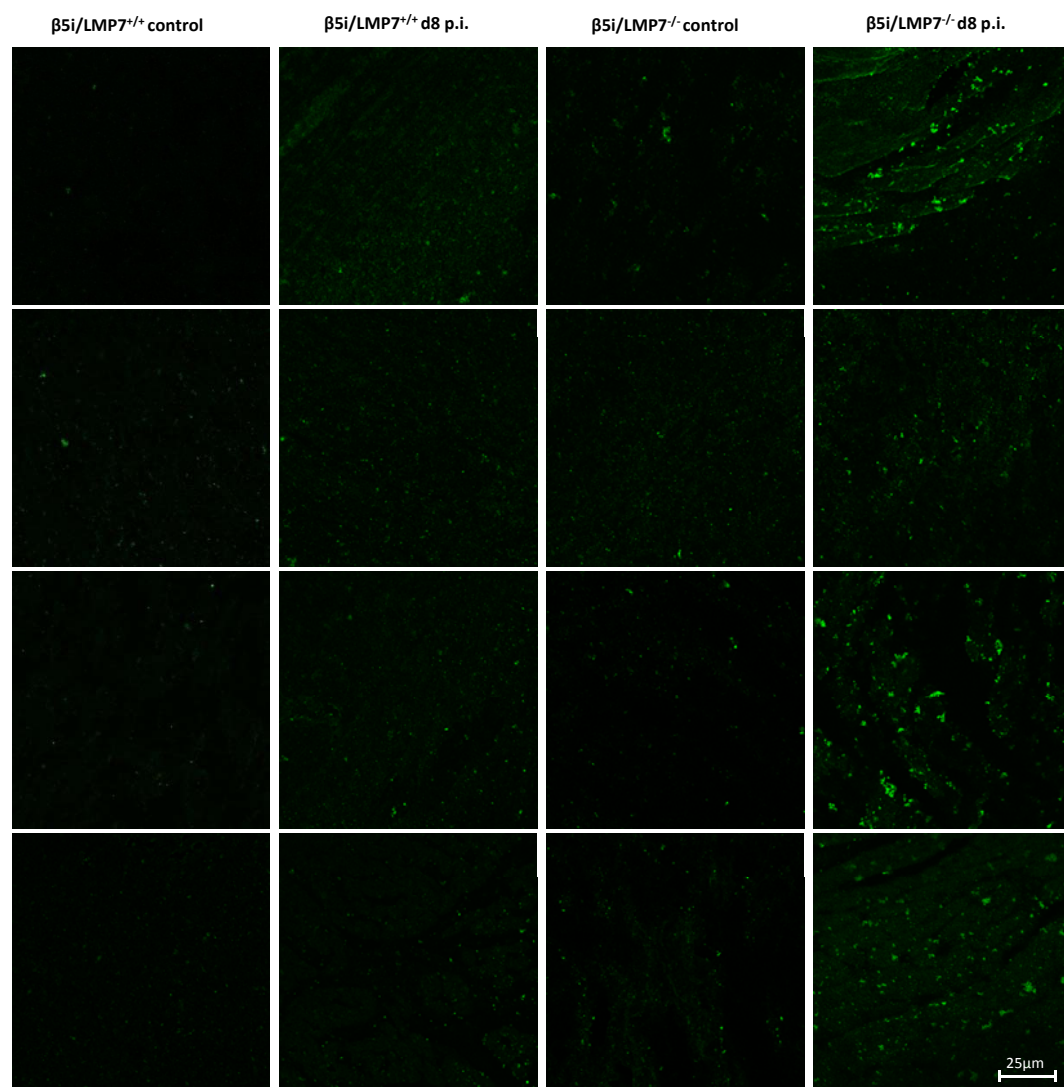

Supplement: Figure S3 (to Fig. 6D) — Accumulation of poly-ub protein conjugates in IP-deficient mice. Formation of ALIS was visualized by immunofluorescence. Heart cryosections from naive and CVB3-infected β5i/LMP7+/+ and β5i/LMP7-/- mice (d8 p.i.) were stained with FK1 for poly-ub (green). Slides from four different individual mice are shown (representative for n = 6 mice from two independent experiments). (PDF) [file ppat.1002233.s003.pdf]

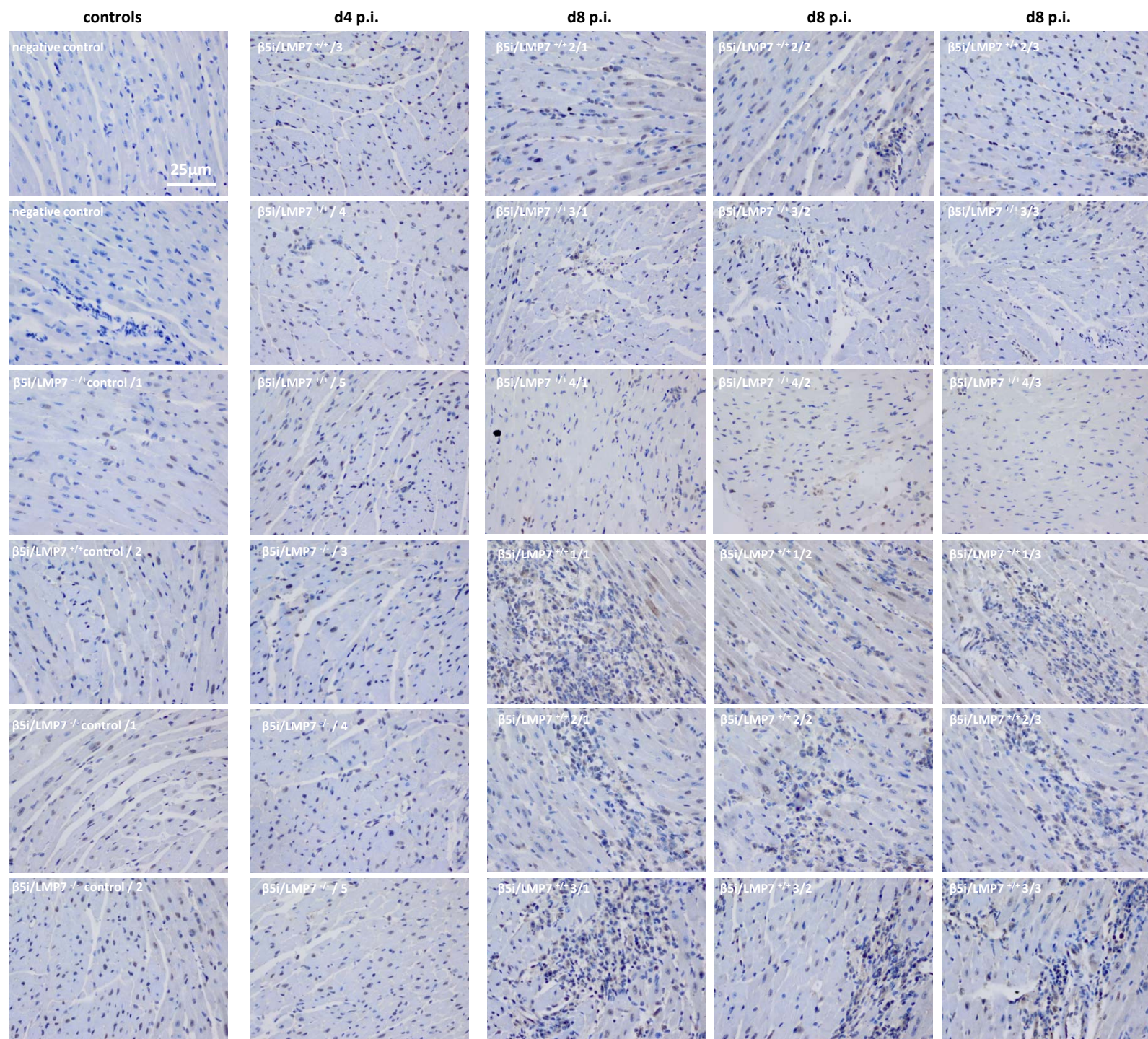

Supplement: Figure S4 (to Fig. 6E) — Accumulation of poly-ub protein conjugates in IP-deficient mice. To visualize poly-ubiquitin conjugates within the injured myocardium, immunohistology staining of ubiquitin was performed in control and CVB3-infected mice (sacrificed at d4 and d8 p.i.). 1st column controls: negative controls reflect secondary antibody staining only within the myocardium (upper panel) and within an inflammatory focus (lower panel). Ubiquitin staining is shown for two representative naive mice from β5i/LMP7+/+ and β5i/LMP7-/- mice. 2nd column d4 p.i. Ubiquitin staining is shown for three different mice from each host. No specific increase in ubiquitin signals is visualizable at this time point. 3rd–5th column d8 p.i. Ubiquitin staining is illustrated for three different mice representing at least n = 5 mice / host. Three different tissue sections are shown per mouse indicating increased poly-ub detection in CVB3-infected β5i/LMP7-/- mice. (PDF) [file ppat.1002233.s004.pdf]

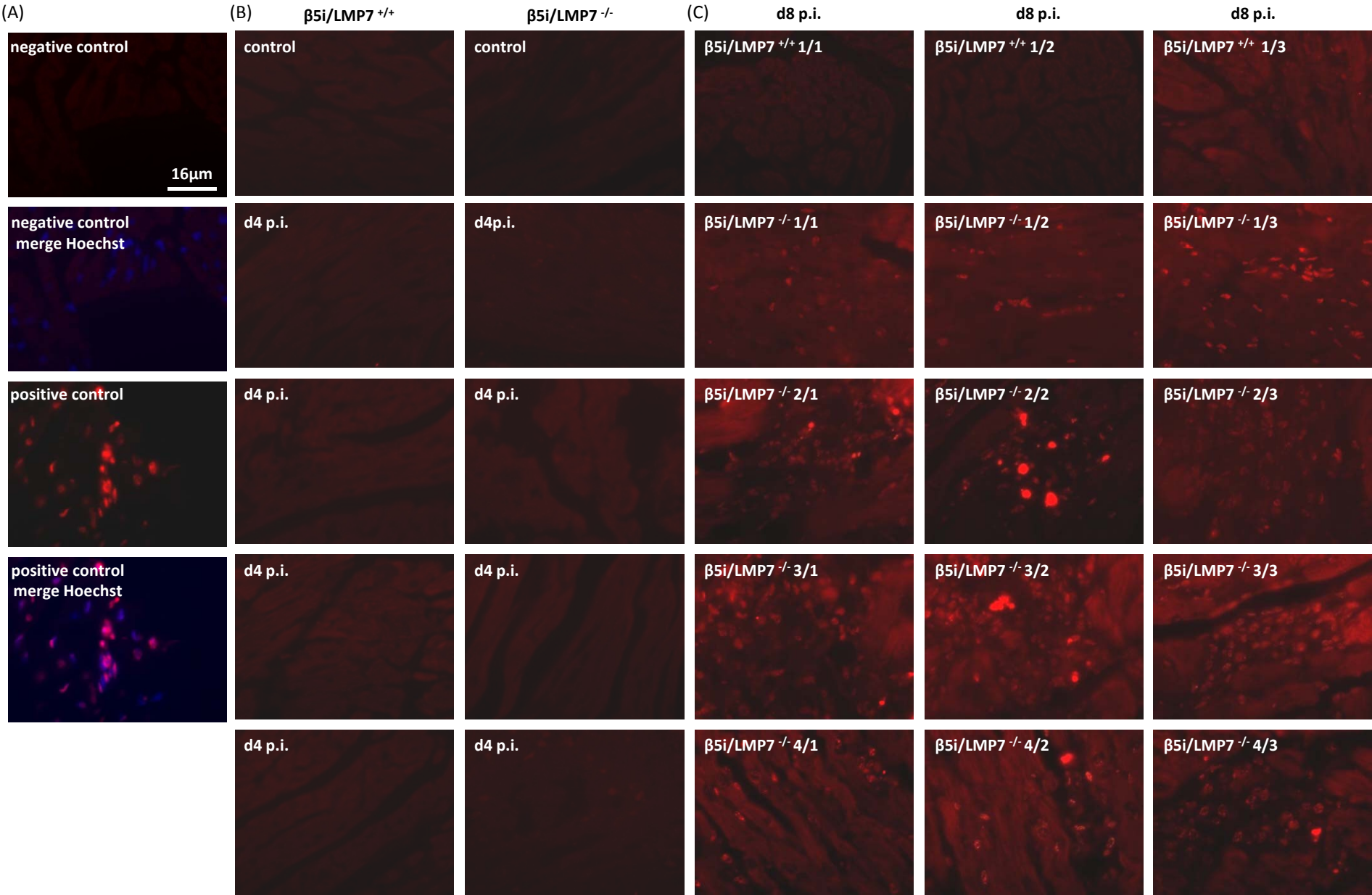

Supplement: Figure S5 (to Fig. 7) — IP-deficient hearts are prone to apoptotic cell death. Apoptosis was assessed in vivo in cardiac tissue sections in naive and CVB3-challenged mice (d4 and d8 p.i.) by in situ cell death detection kit, TMR red. Representative cardiac sections from one independent experiment are shown. (A) Controls: lower two slides illustrate DNA strand breaks within inflammatory lesions and surrounding cardiomyocytes, which colocalize to nuclei (last slide). (B) TUNEL staining is shown for one control heart and four individual mice sacrificed at d4 p.i. (representative for n = 6 mice). No apoptotic cell death was detected in these mice. (C) DNA strand breaks were visualized in CVB3-infected hearts from β5i/LMP7-/- mice: three representative slides are shown for each heart. Increased levels of TUNEL-positive cells are located particularly within inflammatory lesions and surrounding cardiomyocytes. In CVB3-infected β5i/LMP7+/+ mice hardly any apoptotic lesions were detected (1st row representative for n = 8 mice). (PDF) [file ppat.1002233.s005.pdf]

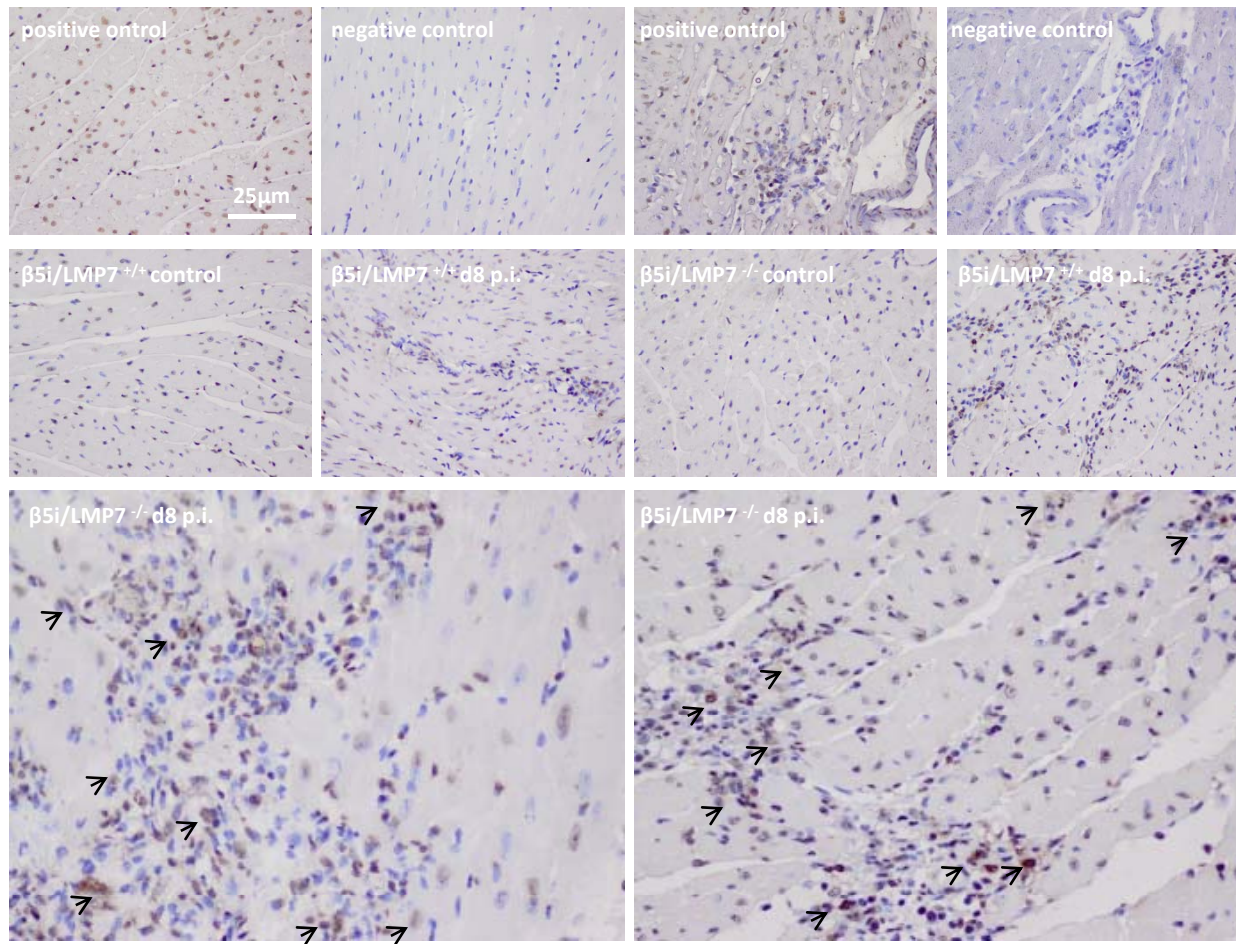

Supplement: Figure S6 (to Fig. 7) — Apoptotic cell death within inflammatory lesions in IP-deficient mice. To further localize cellular injury within the injured myocardium, in situ cell death detection kit, POD was used. Tissue sections are representative for at least n = 5 mice. (PDF) [file ppat.1002233.s006.pdf]
